# Recent consumer OLED monitors can be suitable for vision science

**DOI:** 10.1167/jov.25.2.11

**Published:** 2025-02-26

**Authors:** Tarek Abu Haila, Korbinian Kunst, Tran Quoc Khanh, Thomas S. A. Wallis

**Affiliations:** 1 Centre for Cognitive Science, Institute of Psychology, Technical University of Darmstadt, Darmstadt, Germany; 2 Laboratory of Adaptive Lighting Systems and Visual Processing, Technical University of Darmstadt, Darmstadt, Germany; 3 Laboratory of Adaptive Lighting Systems and Visual Processing, Technical University of Darmstadt, Darmstadt, Germany; 4 Centre for Cognitive Science, Institute of Psychology, Technical University of Darmstadt, Darmstadt, Germany

**Keywords:** vision science, color, luminance, perception, display, calibration

## Abstract

Vision science imposes rigorous requirements for the design and execution of psychophysical studies and experiments. These requirements ensure precise control over variables, accurate measurement of perceptual responses, and reproducibility of results, which are essential for investigating visual perception and its underlying mechanisms. Because different experiments have different requirements, not all aspects of a display system are critical for a given setting. Therefore, some display systems may be suitable for certain types of experiments but unsuitable for others. An additional challenge is that the performance of consumer systems is often highly dependent on specific monitor settings and firmware behavior. Here, we evaluate the performance of four display systems: a consumer LCD gaming monitor, a consumer OLED gaming monitor, a consumer OLED TV, and a VPixx PROPixx projector system. To allow the reader to assess the suitability of these systems for different experiments, we present a range of different metrics: luminance behavior, luminance uniformity across display surface, estimated gamma values and linearity, channel additivity, channel dependency, color gamut, pixel response time, and pixel waveform. In addition, we exhaustively report the monitor firmware settings used. Our analyses show that current consumer-level OLED display systems are promising and adequate to fulfill the requirements of some critical vision science experiments, allowing laboratories to run their experiments even without investing in high-quality professional display systems. For example, the tested Asus OLED gaming monitor shows excellent response time, a sharp square waveform even at 240 Hz, a color gamut that covers 94% of DCI-P3 color space, and the best luminance uniformity among all four tested systems, making it a favorable option on price-to-performance ratio.

## Introduction

A pillar of vision science is a display system that is both accurate and stable (“Know Thy Stimulus”; Geisler, 1987; Wichmann & Jäkel, 2018). However, because different experiments have different requirements, not all aspects of a display system are critical for a given setting. Therefore, different characteristics could be of more or less importance than others depending on the requirements of the experiment at hand.

The cathode-ray tube (CRT) display has long been considered the “gold standard” for many experiments. Consumer liquid-crystal displays (LCDs), for example, are still considered unsuitable for many high-precision experiments. Briefly, CRT displays operate using an electron beam in a vacuum and a phosphor screen by modulating the electron beam the output luminance is controlled. Color CRTs use three beams, one for each of the RGB primaries, and different phosphor coatings that help in producing the RGB colors. To display a frame, the beams need to project and scan the surface of the screen line by line from top to bottom and left to right (Wheeler & Clark, 1992). In LCDs, the liquid crystal molecules are placed behind each pixel and change orientation, in synchrony or individually, to control the output light. The light in LCDs comes from what is known as a *backlight*, usually white, and is filtered when passing through RGB filters that constitute each pixel to output the desired color (Fujieda, 2023a; Fujieda, 2023b).

Elze and Tanner (2012) discussed various aspects and shortcomings of the LCD technology of that time. They addressed the problem of motion blur in LCDs and the luminance stepping in which the different primaries (R, G, and B) show different response time profiles when making the transition from 0 to 255 (8-bit) owing to the different driving signals to the different primaries. They tested, as well, the response time variability that is reported to be in the range of up to 10 ms and the variation coefficient (relative deviation with respect to the absolute response time calculated by dividing the standard deviation by the mean) amounting to 0.25 on average. These reported values are considered unsatisfactory for many vision science experiments.

CRT displays have, as well, their share of problems. Lagroix, Yanko, and Spalek (2012) pointed out how CRT displays actually suffer from *display persistence*, residual image, whereas the tested LCD counterparts showed no such effect. Residual image in CRT displays is the result of the phosphor persistence that remains visible for some time after the image has been turned off. They made it clear that concerning the response time of a pixel (rise and fall), LCD displays have come a long way thanks to overdrive technology—overdrive helps in speeding up the liquid-crystal transition from one state to another (i.e., one brightness level to another) by applying higher voltage. It helps to achieve a faster response time and to eliminate the chance of the ghosting effect. The authors showed a substantial improvement by hitting 1 to 6 ms for the rise time and less than 2 ms for the fall time, beating the stigma around LCDs for having a sluggish response that was reported in various papers in the year 2007 and before. Klein, Zlatkova, Lauritzen, and Pierscionek (2013) pointed out one of the major shortcomings in discussing the inhomogeneity (i.e., non-uniformity) in luminance across CRT displays. Their measurements showed up to 10% variation across the display surface, whereas other authors reported up to 20% variation (Bohnsack, Diller, Yeh, Jenness, & Troy, 1997; Krantz, 2000). Over the subsequent years, CRT displays have become increasingly rare, phased out, and difficult to find owing to advancements in various consumer display technologies, meaning that they are rarely, if ever, commercially available.

In contrast, organic light-emitting diode (OLED) display technology has become more affordable and abundant on the market in recent years. OLED displays are composed of self-emissive diodes that allow the control of pixels independent of one another and eliminate leakage caused normally by the use of a backlight (e.g., in LCDs). This means that, in theory, OLED pixels can produce truly zero luminance, and also that pixels should be completely independent (switching a certain pixel on and/or off would not affect the luminance value of other pixels). These properties of the display technology mean that the contrast range ($\frac{max\;white\;luminance}{min\;black\;luminance}$) of these monitors can approach infinity. They can also offer a larger color gamut thanks to their saturated primaries and narrow-band spectra in comparison with LCD and CRT displays. Thanks to the Helmholtz–Kohlrausch effect, more saturated colors are perceived to be brighter (i.e., an increase in color saturation increases the perceived brightness; Tsujimura et al., 2007; Tsujimura, 2017). Because the perception of brightness is also linked to and affected by the surround colors/ambient light (Bartleson & Breneman, 1967), the same color on a darker background can be perceived as brighter. According to Hack et al. (2010), various psychological and perception studies have reported that thanks to the OLED pitch-blackness ability, users have reported an increase in perceived brightness at the same level of luminance as their counterparts LCD displays. Finally, previous measurements from Elze, Taylor, and Bex (2013) showed that OLEDs are capable of producing extremely precise, almost-square wave, temporal response profiles. Together, these results suggest that OLED technologies hold much promise for use in vision science.

Several previous papers have investigated the suitability of OLED displays for vision science applications. Cooper, Jiang, Vildavski, Farrell, and Norcia (2014) presented a thorough investigation of the suitability of OLED monitors for vision science research and concluded that they are a favorable option. They included in their study two OLED monitors, namely the Trimaster EL BVM-F250 Master Monitor (BVM), a high-end professional video monitor for production applications, and the Trimaster EL PVM- 2541 Picture Monitor (PVM), a video monitor designed for general use. They showed that OLED displays can have a nice additivity property and a wide color gamut and that gamma functions can be fitted to a power function, making their luminance output linearizable if desired. One of the tested OLED showed, as well, pixel independence in the sense that the behavior of vertical pixels is not affected or influenced by horizontal pixels and vice-versa. Elze et al. (2013) tested a very specific medical OLED monitor, namely, the Sony PVM 2551MD, and whether it complies with the digital imaging and communication in medicine (DICOM) standards. They investigated the discretization of the color channels and showed that approximately 50% of the neighboring bit values are perceptually indistinguishable (based on the just noticeable difference defined in the DICOM standards) for the blue channel and approximately 28% for the green channel, rendering the effective perceptual luminance resolution of the monitor to be considerably below it is actual 8-bit resolution. They showed as well that activating all the monitor’s pixels, a full-screen color patch, results in early saturation in contrast relative to a small color patch. For instance, the white color patch showed saturation in full-screen mode as early as approximately 162 cd/m^2^ (corresponding with a bit value of 196) as opposed to the monotonic increase in luminance while increasing the bit value for a small circular color patch that reaches eventually approximately 400 cd/m^2^. In a recent study, Ashraf, Sztrajman, Hammou, and Mantiuk (2024) present measurements of an OLED TV using different regression models on how to run color calibration on a four-primary OLED monitor. The monitor they tested showed a saturation effect across all its channels near the upper limit of the bit depth (bit value >700 in 10-bit depth), breaking any possible linear behavior or compliance with a gamma function. Their work focused only on the color calibration aspect and concluded that the performance of the different color calibration models was best for low luminance levels and worst for higher luminance levels. Tian, Xu, and Luo (2019) showed that the Sony A1 OLED TV suffered from a drastic drop in peak luminance for all its color channels and any window size that was more than 10% under the default settings they chose, and hence they fixed the window size for all their measurements to 4%. Yang, Hsiang, Qian, and Wu (2022) showed that there is a drop in luminance for the OLED display of a DELL XPS 15 7590 notebook, when the average pixel level (APL) factor of more than 1%, a drop from approximately 562 to 431 cd/m^2^. Evaluating the PVM-2541 OLED, Ito, Ogawa, and Sunaga (2013) showed that when the filling factor is greater than 40% and the bit value is 220 (8-bit) then the luminance output drops drastically at least approximately 5% and at most approximately 27%. They also measured how changing the background color affects the luminance output by showing that any value above gray 220 (8-bit) for the background causes a drop in the target luminance up to approximately 27% when measuring a small square in the middle of the display, more noticeably for any gray value above 128, but nonetheless even a gray value of 40 showed the same tendency. Finally, Dimigen and Stein (2024) recently published results evaluating a number of different monitors with a focus on the ASUS OLED panel that we also evaluate here. We compare their results to ours more thoroughly in our Discussion section. In general, the results of the study by Dimigen and Stein (2024) agree with our measurements on the overall performance of this monitor, providing some additional confidence in the results we present here.

Several commercial display systems exist that are purpose-built for vision science, notably the VIEWPixx LCD range and/or PROPixx projector (VPixx Technologies Inc., Saint-Bruno, QC, Canada), and the *Display++* LCD range (Cambridge Research Systems Ltd., Rochester, UK). Ghodrati, Morris, and Price (2015) compared five high-end monitors, including those that are targeted to vision science specifically, and discussed their (un)suitability for vision science research. The tested monitors were EIZO FG2421 LCD, VIEWPixx 3D Lite LCD, Samsung 2232RZ LCD, CRS Display++ LCD, and Sony CPD-G520 CRT. They reported their findings regarding luminance perception and viewing angle, luminance uniformity, and temporal dynamics. At low luminance levels, for instance, high variability was an issue for the tested CRT. Although luminance non-uniformity across the display’s surfaces was as high as 20% for EIZO, Samsung, and Display++ systems. All tested LCDs suffered from luminance hysteresis, in which the desired target luminance level is not achieved in a single frame when making the transition from black to white or vice versa. The hysteresis is attributed to the backlight mechanism implemented in all tested displays.

In this article, we investigate the suitability of four display systems for use in vision science. The requirements for a display device depend on the experiment; deciding which aspects are critical is part of scientific training, and not the subject of this paper. Here, we evaluate displays on the following metrics that, we hope, paint a useful picture of how far the OLED display technology has come and how suitable it is to be deployed in vision science. We consider the following display system properties. 1) Channel additivity: defining the relationship between the response of the individual R, G, and B channels and their collective response when they act in synchrony to reproduce grayscale (R=G=B). Linearity/gamma predictability: defining the relationship between the pixel bit value and its luminance output. Output is predictable based on the bit value, meaning that it can be corrected predictably using a lookup table or gamma function. Pixel (in)dependence: Whether the behavior of a subset of pixels is influenced by the state of other pixels. Signal response time: how long it takes a set of pixels to change state. Signal waveform: defining the signal waveform indicating the actual duration pixels take in one state at the desired luminance level before switching to another state. Color gamut: how wide the color gamut a certain display offers (i.e., number of available colors). Luminance uniformity: how homogeneous the luminance output is across the display surface. APL: the relationship between the overall active pixels intensity (bit value) and the luminance output to determine how much the luminance output is invariant to the overall pixel values and proportion of the display using grayscale (R=G=B). Filling factor primaries: same as the APL. However, instead of testing only the behavior of grayscale, we test the RGB primaries’s behavior.

**Figure 1.**
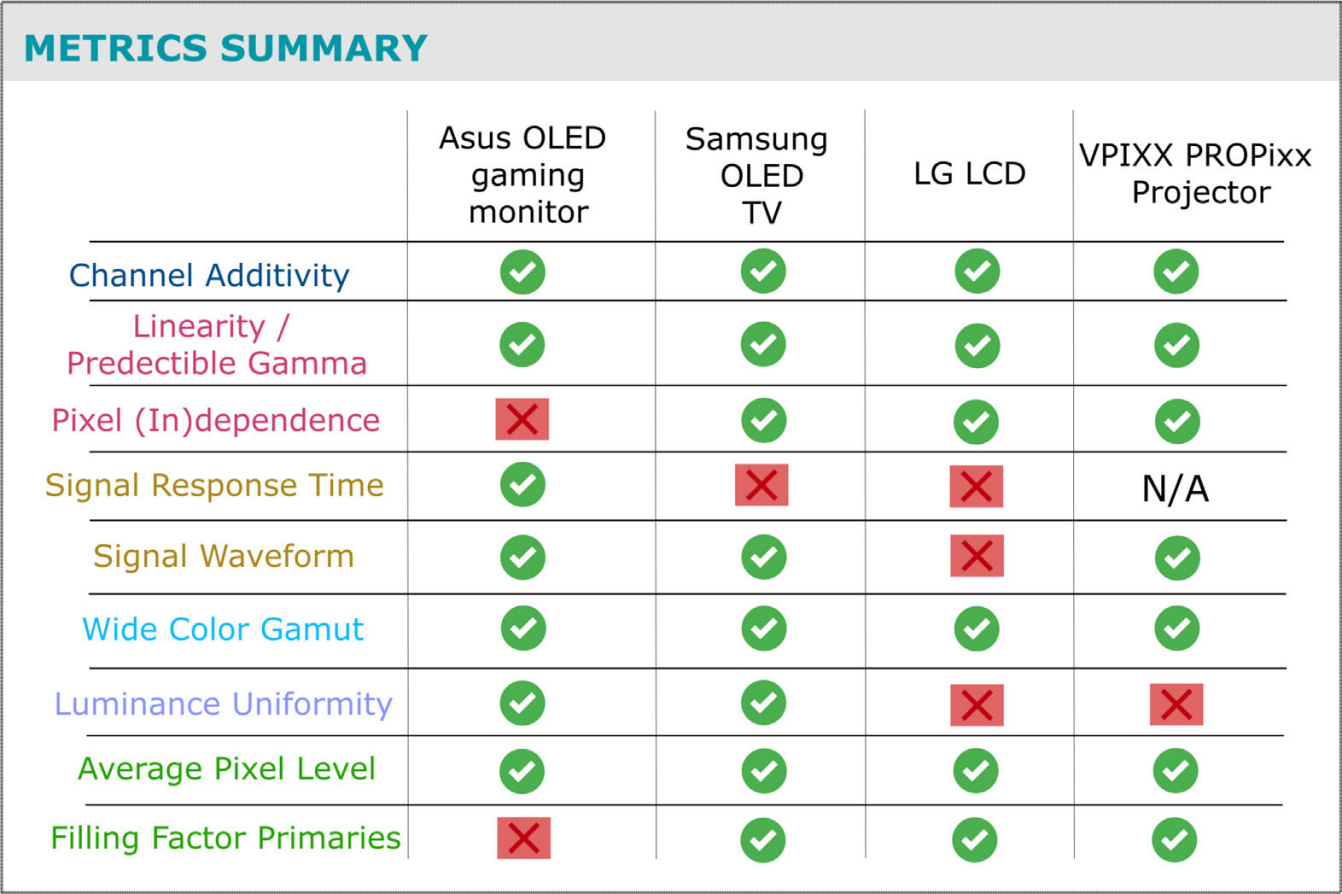
A summary of the performance of the four tested display systems on some of the display properties we consider. The pass–fail indicators should not be interpreted as definitive criteria for the acceptance or rejection of any particular system. Rather, they serve as a means to evaluate whether a system meets its theoretical hardware specifications and operates within expected performance limits.

The display systems we test consist of two OLEDs, an LCD, and an LED projector; these are described in more detail below. Overall, we find that consumer-grade OLED technology is promising as an affordable alternative to LCDs and CRTs, that can still be as reliable and competent in important aspects of performance as some specialized display systems. Figure 1 offers a coarse summary of each tested system’s performance on the different properties we consider.

## Material and methods

### Hardware

#### Display systems

We test four displaying systems: the PROPixx projector (VPixx Technologies Inc.), ASUS OLED ROG Swift PG27AQDM gaming monitor, a Samsung OLED TV GQ65S93CAT, and an LG UltraGear 27GN950-B LED gaming monitor, check Table 1.

**Table 1.** A summary of the display system’s main specifications.

Display	Model	Light technology	Native resolution	Pixel density per mm	Bit depth	Max. refresh rate
Asus	ROG Swift PG27AQDM	OLED	2560 × 1440	4.36	10	240
LG	UltraGear 27GN950-B	White LED	3840 × 2160	6.48	10	144
Samsung	GQ65S93CAT	OLED	3840 × 2160	2.70	10	144
PROPixx Projector	VPX-PRO-5050C	RGB LED	1920 × 1080	1.95	12	1440

The PROPixx projector is designed specifically for vision science applications. It is based on DLP technology and RGB narrow LEDs and can achieve 12-bit depth per channel. Its native resolution is 1920 × 1080 at 120 Hz, and it can operate at frequencies of up to 480 Hz in color and up to 1440 Hz in grayscale at a lower spatial resolution. The projector uses DMD technology for displaying and light manipulation, it uses the AeroView 100 screen (Stewart Filmscreen, Torrance, Canada). Its output is already perfectly linear, so gamma correction is unnecessary. The throw distance between the rim of the lens and the projection screen measured to approximately 92.5 cm and the projected image measured to approximately 97.5 × 54.5 cm and displayed on the AeroView 100 rear projection screen. The measurements of the PROPixx serve as a baseline in this article.

The ASUS ROG Swift OLED PG27AQDM monitor is 27” and uses LG OLED RGBW panel. It offers true 10-bit depth per channel and coverage of up to 94% of the DCI-P3 color gamut. It has a native resolution of 2560 × 1440 and can operate at a frequency of up to 240 Hz. The display can reach a peak brightness of 500 cd/m^2^ under certain settings.

The LG UltraGear 27GN950-B is 27” and uses white LED as a backlight. It has a native resolution of 3840 × 2160 (4K) with an 8-bit panel + FRC (Frame Rate Control) so to achieve 10-bit-like behavior and offers up to 95% coverage of DCI-P3 color space. Its refresh rate can go up to 144 Hz. The maximum luminance of the monitor is advertised to reach up to 400 cd/m^2^.

Finally, the Samsung OLED TV display GQ65S93CAT is 65” and offers a true 10-bit depth with a native resolution of 3840 × 2160 (4K) and can be driven up to 144 Hz. It uses quantum-dot technology as the underlying lighting mechanism.

The difference between the two OLED displays (Asus vs. Samsung TV) is that the Asus OLED uses four subpixels to reproduce colors, namely, the common RGB + an additional white subpixel based on the LG panel *LW270AHQ-ERG2*, which enables the display to reach higher brightness levels. The Samsung OLED TV, on the other hand, uses a blue OLED backlight and quantum-dot technology to produce pure and saturated RGB primaries, which allows the display to have a noticeably larger color gamut and more saturated colors. Quantum dots have the properties of narrow and brighter emission, a higher signal-to-noise ratio thanks to their inorganic composition in comparison with organic dyes. One major difference is that the full width at half maximum (FWHM) of the emission peak is as half as that of organic LED, 20 to 30 nm vs. 50 nm. Hence, better color contrast and saturation (Ugarte, Castelló, Palomares, & Pacios, 2012).

The pixel density of each display in millimeters is calculated by dividing the diagonal pixels over the diagonal physical length in millimeters, as shown by Equation 1. The pixel density besides some other main characteristics of each of the displays are summarized in Table 1

(1) $$\begin{array}{c} PPM=\frac{dp}{dl}=\frac{\sqrt{X^{2}+Y^{2}}}{W^{2}+H^{2}}\; \end{array}$$

where *PPM* is pixels per millimeter, *dp* is diagonal pixels, *dl* is diagonal length in millimeters, *X* is horizontal pixels, *Y* is vertical pixels, *W* is width in millimeters and *H* is height in millimeters.

#### Measuring devices

For measurements, we used the following instruments: A colorimeter from X-Rite (i1Display) for calibrating the monitors and measuring the luminance levels (cd/m^2^). The colorimeter luminance range is 0.1 to 1000 cd/m^2^. An X-Rite spectrophotometer (i1Pro 3) for measuring the spectral power distribution (SPD) curves with operating range of 380 to 730 nm and optical resolution of 10 nm. A response box from OSRTT (OSRTT Pro CS) to measure luminance transition response time and pixels waveform. The response box comprises six photodiode sensors aligned horizontally and occupies a width of approximately 19 mm.

We verified the accuracy and reliability of the colorimeter and the spectrophotometer measurements against a more precise and calibrated instrument, namely a spectroradiometer from Konika Minolta (CS2000). The minimum sensitivity of the CS2000 is 0.003 cd/m^2^ at 1°. OLEDs are capable of switching off their pixels completely, as mentioned elsewhere in this article, to represent black, as well as their capabilities of representing very low luminance levels. As a result, very low luminance levels, less than 0.1 cd/m^2^, were not possible to register using the colorimeter (i1Display), even though some of these luminance levels could be perceptible by the human eye after good dark adaptation.

The measurements for the Asus and LG displays were performed on Ubuntu 22.04 and a control PC with Intel Core i9 14th Gen., 32 GB RAM, and AMD Radeon RX 7800 XT 16 GB. The control PC for the Samsung display was an Intel Core i9 13th Gen. 64 GB RAM, NVidia GeForce RTX 4090, and under Ubuntu 20.04. For PROPixx, the driving PC was an Intel Core i7 11th Gen., 16 GB RAM, and AMD RX6600 XT 8GB, under Ubuntu 22.04. Only the signal response/waveform measurements were measured under Windows OS 11 because the used measuring instrument, *OSRTT Pro CS*, runs exclusively on Windows. It was not possible to run all the measurements on one PC, because each display is in a different location and is used for different purpose. However, all the control PCs run a Linux operating system, and we suspect little to no variation in the measurements owing to the different types of graphics cards, as all of them are high-end and of a similar tier. The experiments were written in Python and the stimuli were generated with the help of *Psychopy* library. All displays except PROPixx were connected via HDMI 2.0 cable at a resolution of 1920 × 1080 at the maximum refresh rate and with 10-bit depth per channel.

Before every measurement session, we checked the system calibration profile for any deviation in peak luminance, gamma, or color accuracy and we recalibrated to the target settings if needed. We left the display to stabilize before recording any measurements at least for 30 to 45 minutes.

Owing to the resolution of the X-Rite i1Display colorimeter (minimum sensitivity that starts at 0.1 cd/m^2^), there were some unregistered luminance values at the beginning of the bit range for the OLED displays that were too dim to be registered by the colorimeter. These values were omitted. It is worth mentioning as well that these instruments, X-Rite colorimeter and spectrophotometer, are contact instruments and hence are mounted directly onto the measured surface of the displays orthogonally. Owing to this constraint, it was not possible to measure the relationship between the output luminance and the viewing angle.

### Metrics

In our test, we devised the following metrics that would offer a good insight into the capability and the suitability of the displaying systems under the test.

#### Spectral power distribution

The SPD defines the spectral profile of the light sources used in a display along the visible domain, usually 380 to 780 nm, and paints a picture of how efficient these primaries are at mixing colors which defines the size of the color gamut eventually. Narrower spectral curves mean more saturated primaries, corresponding to a larger color gamut. For these measurements, we used the X-Rite i1Pro 3 spectrophotometer.

#### Luminance response

The luminance ramp is the transition behavior of each channel along the available bit depth. We measure each of the primaries (R, G, and B) as well as the grayscale (i.e., R=G=B). All the luminance measurements are taken from a small patch (512 × 512 pixels) in the center of the display against a mid-gray background unless stated otherwise. The luminance transition covers the whole available bit depth (10 or 12 bits), depending on the capabilities of the tested display. The measurements were done in bit steps of one or five. Five-step transition was used only for the 12-bit depth of PROPixx because the measurements were time-consuming (even with five-step transition one-channel measurement would still take up to 50 minutes). All other measurements were done at one-step transition.

#### Pixel (in)dependence

Pixel (in)dependency is a measure to check how the luminance output of a certain group of pixels would be affected by the behavior of another group of pixels at a different region of the display. We verified this in two ways: 1) While keeping the measured color patch at the center and only changing the background color from mid-gray to black and 2) measuring a small patch in the center on a mid-gray background while fixing two primaries to the maximum while varying the third one, for example, [*R*_*max*_, *G*_*max*_, *B*_*DDL*_] (DDL: digital driving level or bit value). We referred to the second scenario as *other channels saturated* (*OCS*).

#### Channel additivity

Additivity checks whether the response of the individual RGB channels sums up to the response of the channels when they act collectively, Equation 2, to produce a grayscale (black to white). Hence, a channel output is invariant and independent of the state of other channels, at the same time it has the same output when combined with other channels to act in synchrony.

(2) $$\begin{array}{c} \;R_{n}+G_{n}+B_{n}\overset{?}{=}{\left(RGB\right)}_{n}\;\;\forall \;\;n\in \left[0-\left(2^{bit}-1\right)\right]\; \end{array}$$

#### Linearizablitiy and gamma predictability

The linear response of a system is a very important property for various psychophysical experiments, for a linear pixel response ensures that a generated stimulus at a certain luminance level is capable of generating double the luminance when the pixel value of that stimulus doubles. All displays have what is known as a *gamma function* (γ), which is, in its simplest form, a non-linear power function that relates between the input value (*V*_*in*_) and the output value (*V*_*out*_) Equation 3 (Bertalmío, 2020; Pelli & Zhang, 1991; Poynton, 1998).

(3) $$V_{out}=V_{in}^{\gamma }$$

A good display system would either show a perfect linear behavior or a predictable and steady gamma behavior giving the possibility for linearization upon decoding the applied gamma value. Because the PROPixx projector is designed with vision science in mind, it comes with a perfect linear behavior for all its channels (RGB). However, commercial displays come with various gamma functions depending on the purpose of viewing (e.g., cinema, bright surround, dark surround, HDR, gaming).

In case of a deviation in the gamma encoding values across the individual RGB channels, it is advised to control and decode each channel separately with its precise value to ensure a perfectly linear behavior of each channel. The encoded gamma behavior of each channel is determined by the ICC calibration profile, besides the behavior of the display hardware itself.

#### Color gamut

Color gamut is defined, usually, by a triangle on CIE 1931 xy-chromaticity diagram, the tips of this triangle are the xy-chromaticity coordinates of each of a display’s RGB primaries at their maximum and acting separately (Ohta & Robertson, 2005; Tooms, 2015). Each color gamut also has a white-point (WP) that defines the chromaticity of the color *White*. It determines how all other colors are rendered relative to this CCT (Berns, 2019). A common WP for displays is a D65 (6500 *Kelvin*).

Keep in mind that a color gamut is a three-dimensional representation of the colors, whereas CIE 1931 diagram is only two-dimensional, with the assumption of fixing the luminance level to a certain point. Hence, the luminance dimension is missing. Both the luminance capabilities and the spread of the primaries define how large and rich a color gamut is, along with the usable bit-depth defined by the display.

#### Luminance uniformity

Spatial uniformity is a metric that ensures that the luminance output is invariant to its location on the display surface. We divide the displays into a 3 × 3 grid and measure the grayscale of each section individually.

#### Filling factor

Historically people used the term *average picture level*; however, it has been suggested to be replaced with *APL* for it is more accurate (Poynton, 2012). The APL is expressed as a percentage of the maximum brightness of a display as a function of the averaged bit value which is the average intensity of all active pixels across a display. APL always uses a white patch on a black background for evaluation, so when *APL* = 0% means all pixels are black, *APL* = 50% means one-half of the display is white, and *APL* = 100% all pixels are white. We analyze the grayscale in addition to each of the primary channels individually (R, G, and B). For this reason, we will use a more generic term like the *filling factor* to express the percentage of the active pixels of a certain bit value across (R, G, B, and grayscale) beyond being restricted only to a white patch. We mention as well the state of the background pixels to be either black, white, or mid-gray. This thorough analysis of the different combinations will provide a detailed insight into the behavior of the primary channels concerning the average intensity of the active pixels.

Many display technologies, inherently, have a built-in safety mechanism known as *automatic brightness limiting* (ABL), which is triggered proportionally to the APL value to limit and protect the hardware from high power consumption, pixels aging, and heating. Some displays may offer an option that disables or limits the action of ABL, however, at the expense of the achievable peak luminance.

#### Signal response time and waveform

The response time is measured for each display using the gray-to-gray concept, which measures the time it takes pixels to transition from one grayscale value to another (International Committee for Display Metrology, 2023b).

We measured the waveform of exactly 1-frame and 11-frames, transitioning from black to white (0.0 → 1.0), within 1 second for each of the displays. The duration of a frame is a function of the refresh rate, $\frac{Time}{Refresh\;Rate}$. For example, for a 240 Hz display, a frame should last 1000/240 = 4.16 ms. A good waveform would be an instantaneous rise and fall of the signal on demand so that the target luminance level is reached exactly when called so as to appear exactly at the beginning of the frame and vanish with its sharp transition back at the end of the frame so one has the stimulus visible for exactly the whole frame period with no retention effect in the best case scenario.

### Calibration

All tested displays, except PROPixx, were color-calibrated using DisplayCal calibration software and the i1Display colorimeter before recording any of the measurements. The software allows the user to specify the parameters of calibration manually if desired. We chose to calibrate with the following parameters for the three commercial displays: max luminance: 250 cd/m^2^ for Asus OLED & LG LCD, 192 cd/m^2^ for Samsung OLED TV; color temperature: 6500 K; tone curve: Gamma 2.2; and calibration speed: high (approximately 16 minutes). PROPixx offers several sequencer modes in which it controls the output color (RGB vs. grayscale) and the refresh rate (120, 180, 240, 480 Hz). VPixx support advised us to choose *RGB 120 Hz Calibrated high bit depth* sequencer to target accurate luminance representation, although this mode drops the maximum luminance to approximately 128 cd/m^2^.

Full specifications of the monitor settings we used for each display are provided in the Appendix.

## Results

### Spectral power distribution

**Figure 2.**
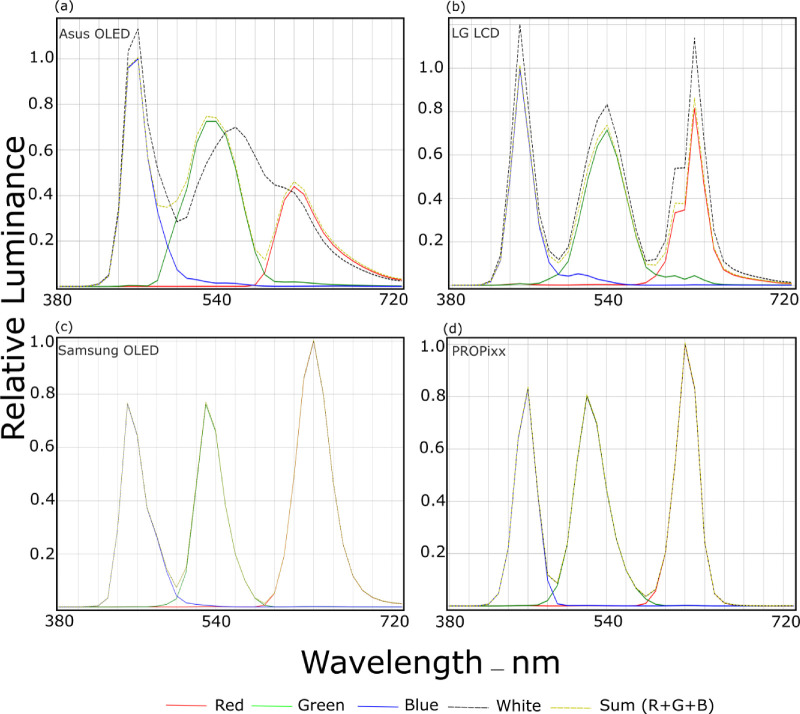
Spectral power distribution (SPD) curves of the four tested displays. The colors of the curves correspond with the measured channels RGB accordingly. In addition, the black curve represents the measured white color SPD, and the yellow curve represents the theoretical summation of the primaries’ SPDs (R+G+B). (a) Asus OLED, (b) LG LCD, (c) Samsung OLED TV, and (d) PROPixx. This arrangement of subfigures is followed throughout this paper in this order. The vertical tick spacing on the x-axis corresponds with a step of 20 nm.

In Figure 2, the four display’s SPDs are plotted side by side for each of their primaries (R, G, and B) channels plus a dashed curve representing the white SPD. Narrower curves and less overlap are favorable as that indicates more reproducible color variations. Also if a system has a perfect additive property one should notice that by looking at how the white SPD behaves in an RGB three-primary system. That is, the white is a result of the activation of all used primaries—talking strictly about three-primary systems—then the white spectral curve would overlap perfectly with the separate RGB channels’ curves, which is the case for both the Samsung OLED TV and the PROPixx projector (Figures 2c and 2d, respectively). For LG LCD, the white SPD seems to have a perfect overlap over its primaries however a little bit amplified in magnitude, whereas for Asus OLED, it is clear that the white SPD does not look like its the exact summation of its RGB primaries (Figure 2a), keeping in mind that this Asus OLED model uses a four-primary system RGBW. One of the purposes of having a white subpixel in such systems is to increase the display’s brightness range. In addition to that the additional white subpixel plays a role in reducing power consumption (i.e., it can cut power consumption by half compared to RGB-subpixel systems).

Also, it enlarges the color gamut and hence represents more pure colors (OLE, 2017). It is worth noting that Chesterman, Piepers, Kimpe, De Visschere, and Neyts (2016), in their work, found heuristically that driving the pixels to their white point with maximum pixel value triplet (255, 255, and 255 in an 8-bit system) activates not only the white subpixel, but also the red and the blue subpixels. However, they found experimentally a triplet of (252, 255, and 215) that would activate only the white subpixel. As to why the Samsung OLED TV behaves differently from Asus OLED, we know that hlthe Asus OLED monitor uses the underlying LG LW270AHQ-ERG2 panel which is RGBW. In contrast, Samsung OLED TV uses Quantum Dot (QD-OLED), which mainly relies on a blue back-light with quantum dots layer and red and green filters to output RGB colors. The quantum-dots technology contributes to enhancing the display’s primaries and having narrow-band spectral curves for the primaries, hence a larger color gamut as one can observe by comparing the curves of Samsung vs. Asus OLED systems (Figures 2c vs. 2a, respectively).

### Luminance response

**Figure 3.**
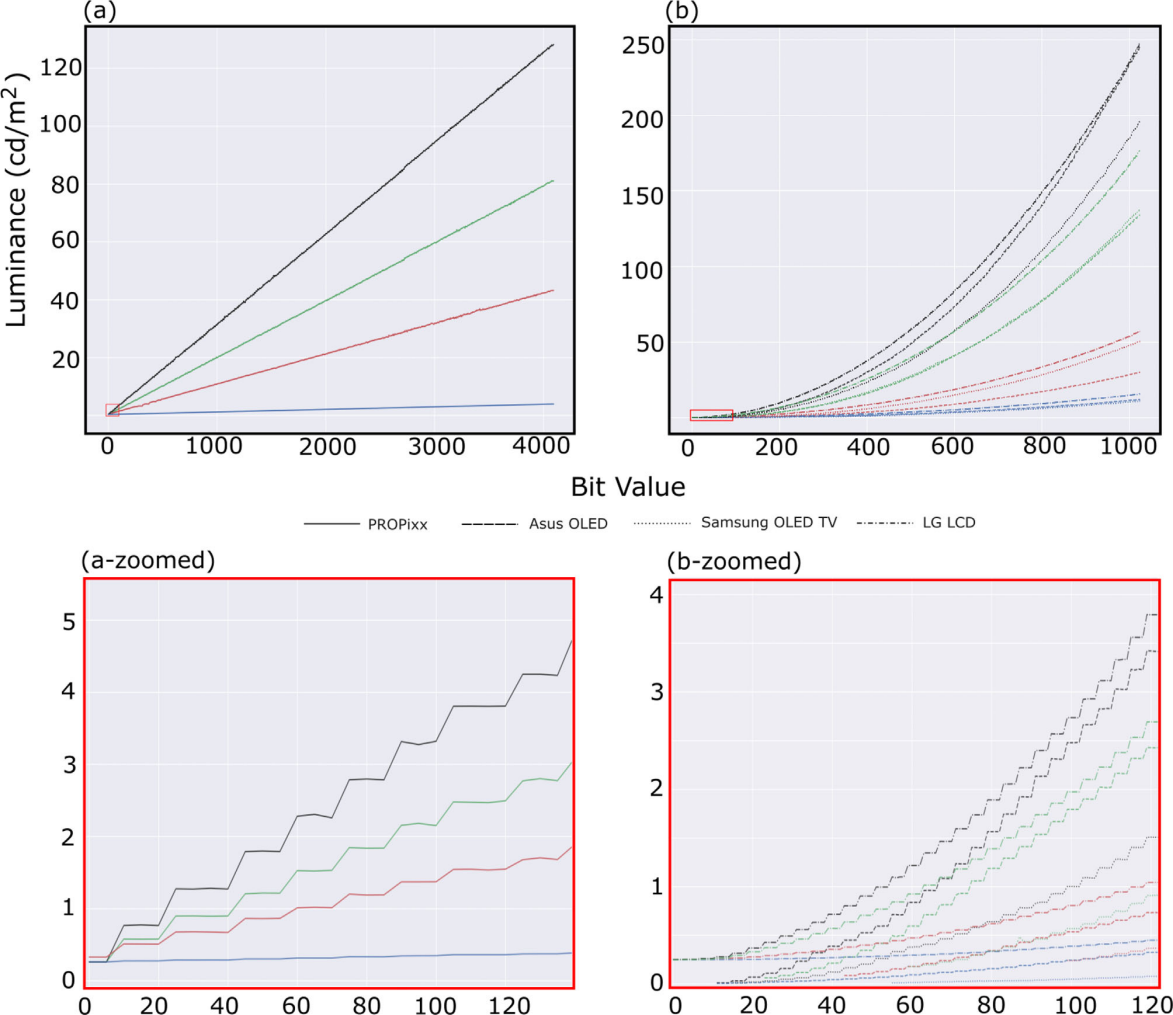
Luminance ramps of RGB channels plus grayscale of the four display systems, (a) PROPixx, (b) contains Asus OLED (dashed), LG LCD (dash-dot), and Samsung OLED (dotted). The curves represent the measured channels RGB with their corresponding colors, grayscale is in black. The three display systems (Asus OLED, Samsung OLED TV, LG LCD) have a similar behavior and do not show any major difference in this test. The PROPixx projector is perfectly linear as expected. The figures in the bottom row show a zoomed-in portion of each of the two figures for luminance levels of less than 5 cd/m^2^. Pay attention to the x-axis that is not unified.

Figure 3 shows the luminance ramps of the RGB primaries and grayscale. The measurements of Asus OLED are plotted in dashed lines, the LG LCD’s are in dash-dotted lines, and the Samsung OLED TV’s are in dotted lines (Figure 3b), and the measurements for PROPixx are plotted separately on the left subfigure (Figure 3a). The colors of the curves match the measurements of the corresponding RGB channels and the black curves for the grayscale measurements. The general behavior of all three displays (Asus OLED, Samsung OLED TV, and LG LCD) seems to be very similar to one another. PROPixx is calibrated to behave in a linear fashion by default, as can be seen from Figure 3a. Changing the background color from mid-gray to black did not show any sign of difference that was worth the attention on the tested displays. It is worth mentioning here that in Ashraf et al. (2024) discussing a different OLED model, namely LG 27” UltraGear 27GR95QE which is also an RGBW system, their measurements of a small patch on a black background showed a sign of saturation for all channels at a certain bit value, nearly more than 700, in the upper half of the 10-bit range.

**Figure 4.**
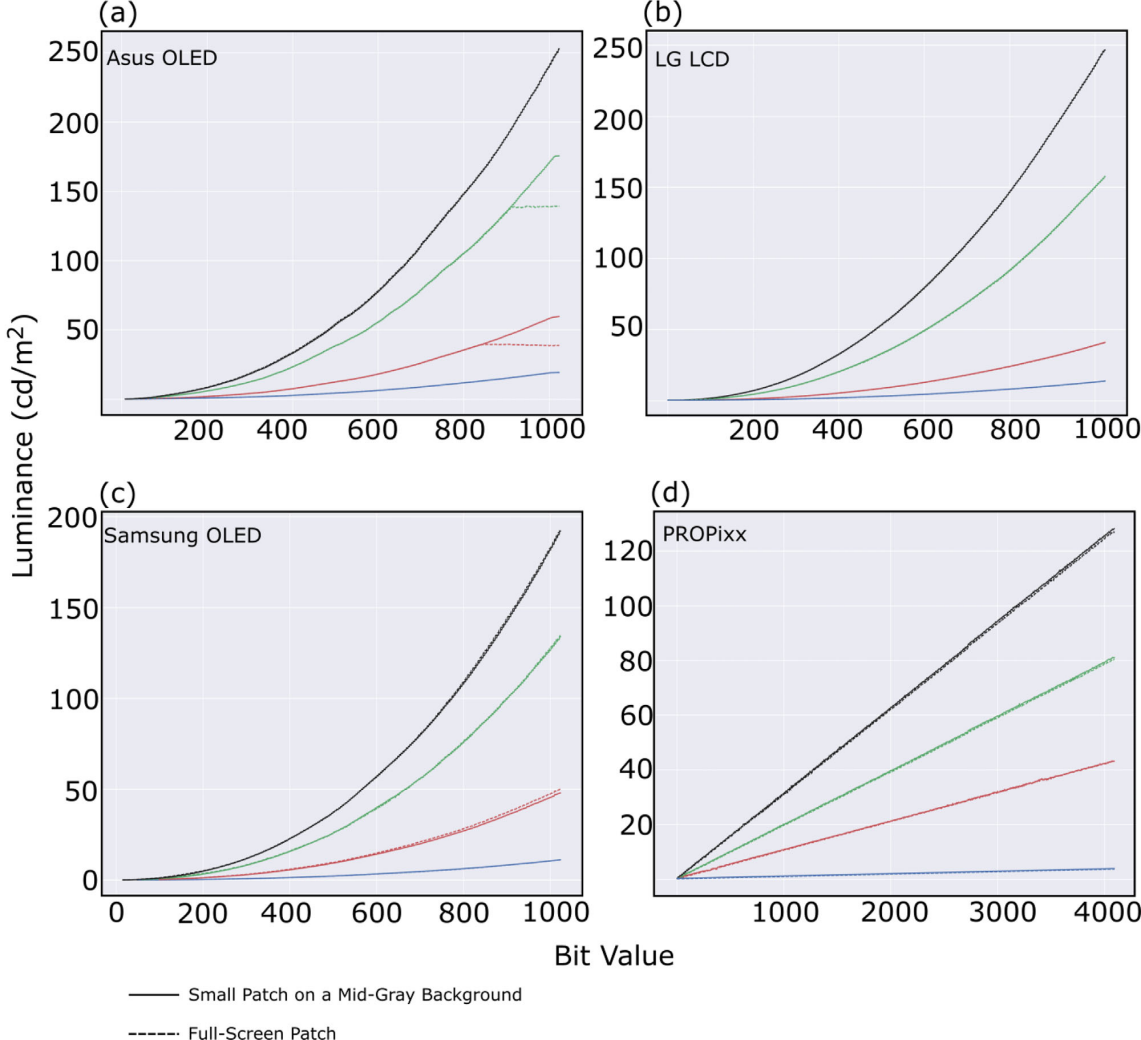
Luminance ramps of RGB channels plus grayscale of the four display systems, (a) Asus OLED, (b) LG LCD, (c) Samsung OLED TV, and (d) PROPixx. The luminance curves show two different tests, solid lines represent small color patch (512 × 512) on a full-screen mid-gray background versus dashed-lines represent full-screen color patch behaviors. Note the unequal x- and y-axes between the panels.

We repeated the test, however, this time with the color patch filling the display, full-screen color, activating all the display’s pixels to act in synchrony. Figure 4 shows the results in 4 subfigures one for each display system, (a) Asus OLED, (b) LG LCD, (c) Samsung OLED TV, and (d) PROPixx projector. The curves in the figure take on the same color as the measured channel, in addition to black for the grayscale. The solid lines show the same test result as in Figure 3 (small color patch on a mid-gray full screen), while the dashed lines are for the full-screen color patch measurements. The Asus OLED monitor stands out among all four tested displays for its behavior in full-screen mode – In Figure 4a the green channel starts to saturate at a luminance level near 138 cd/m^2^ and pixel value of 916 in a 10-bit range in comparison with the maximum green channel luminance for a small patch at 175.5 cd/m^2^ , and the red channel starts to saturate around luminance level of approximately 39 cd/m^2^ around pixel value of 849 in comparison to maximum luminance level of 59.6 for a small color patch. The blue channel’s and the grayscale’s behaviors stay unaffected. We noticed that, if we adjust some of the six-axis saturation settings, the overall display saturation goes down and the effect of ceiling gets resolved, however on the expense of other metrics like the additivity breaking down. The saturation behavior seems to agree with the findings of Chesterman et al. (2016) for the same type of OLED (RGBW) that states that when all the pixels are active then luminance saturation of RGBW OLED is inevitable at higher bit value—also known as DDL. Both the LG LCD and the Samsung OLED TV use backlight but completely different technologies for producing RGB subpixels, and neither of them shows any sign of limitation similar to Asus OLED for reaching the maximum luminance while driving all the display’s pixels at once—full-screen mode. PROPixx projector, as well, does not suffer any limitation in this regard.

**Figure 5.**
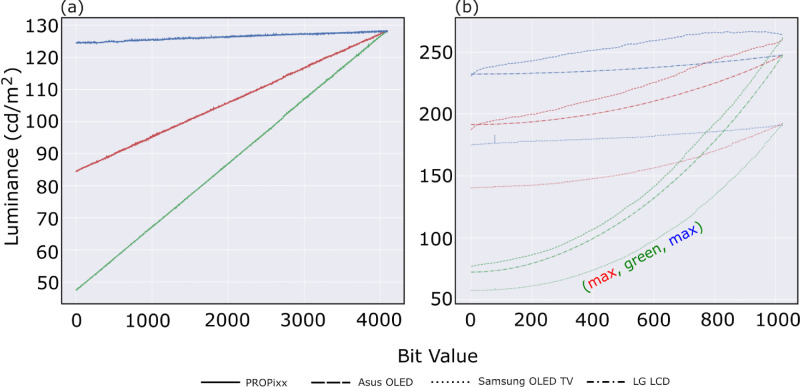
Luminance ramps of RGB channels, when two channels are saturated and one is ramping up, for ASUS OLED monitor (dashed), SAMSUNG OLED TV (dotted), and LG LCD monitor (dash-dot). The colors of the curves match the channel that is varying in value.

While measuring OCS, we noticed that for ASUS OLED the maximum luminance exceeds the maximum luminance level when measured at the end of the grayscale ramp. The blue channel is very noticeably overreaching the calibrated maximum luminance, which is 250 cd/m^2^, as can be seen from the results in dashed lines for the blue channel in Figure 5b. This should be understood in the light of Chesterman et al. (2016) study, for in RGBW OLED systems the maximum white is usually not a result of just mixing the common three primaries RGB, nor just activating the white subpixel by itself. Rather, it is a combination of white and other subpixels in action so that the display outputs more luminance. On the other hand, for Samsung OLED TV (dotted curves), the OCS behavior is more steady except for one abrupt spike that occurs around a bit value of 77 during the measurements, we could not explain why this happened even in repeated measurements. The spike happens across all channels and is the strongest in the blue channel which amounts to a change in the luminance level from approximately 176 to 183 cd/m^2^ a change of nearly 4%. Note also the maximum luminance level for Samsung OLED TV of approximately 192 cd/m^2^ in comparison with LG LCD and Asus OLED that is approximately 250 cd/m^2^. PROPixx, Figure 5a behaves very linearly as expected. LG LCD and PROPixx did not show any behavior that was worth the attention.

### Channel additivity

**Figure 6.**
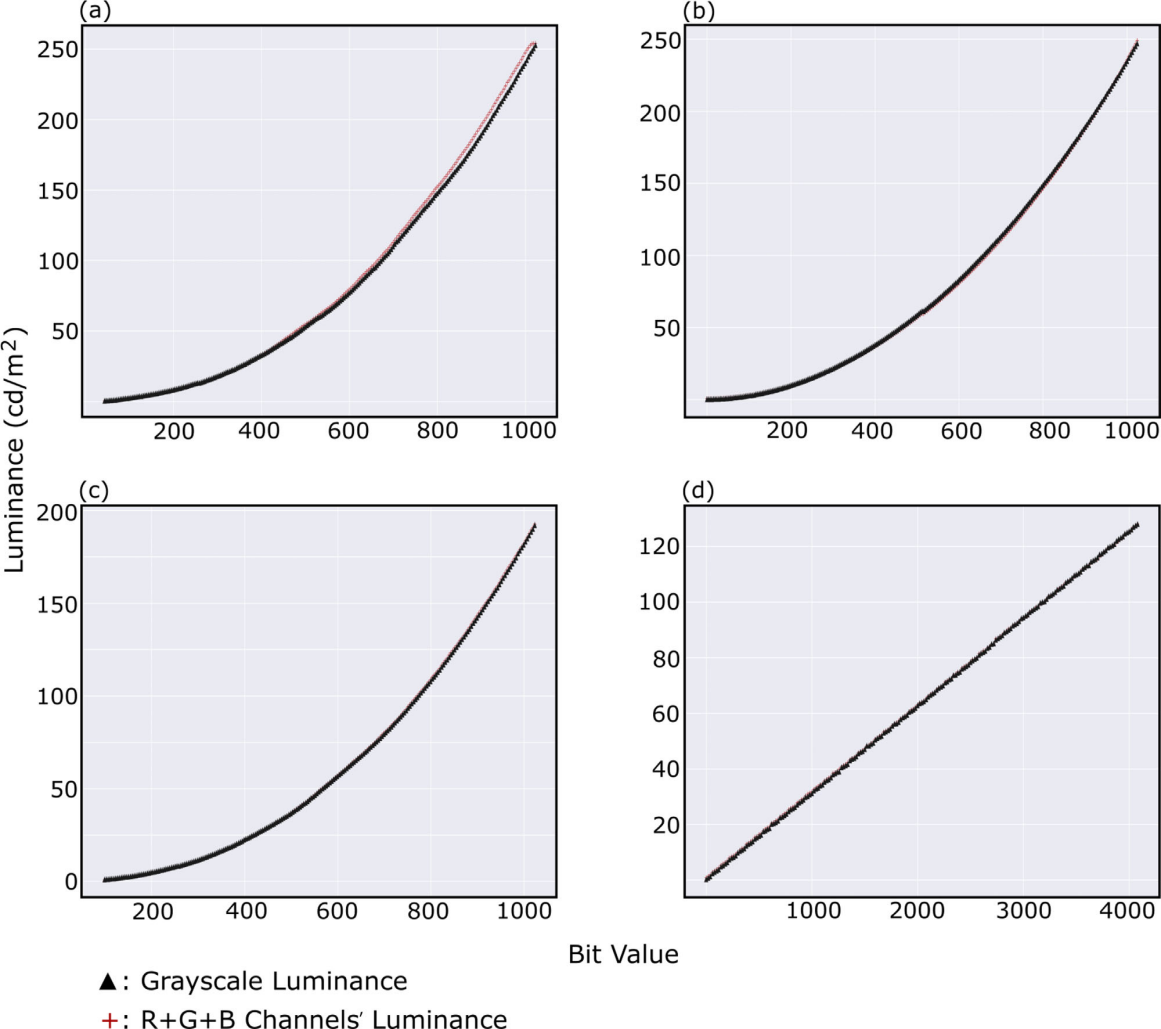
Additivity test showing the luminance levels of the summed RGB primaries at a certain bit value (‘+’ sign) and the corresponding luminance level for the same bit value along the grayscale (‘▲’ sign).

Table 2 shows the exact values for the white luminance versus the summed maximum luminance of the individual RGB channels showing that a linear behavior governs all four display systems under the test. Figure 6 illustrates the additive behavior all along a grayscale (black to white) in which the ▲ sign represents the luminance level of a grayscale value (i.e., subpixels acting together), while the ‘+’ sign represents the summation of the luminance levels at that value when the RGB channels were measured individually. All four display systems show a congruent behavior in which the RGB channels act individually in the same way as when they act collectively to produce a certain luminance level under the chosen settings. One notices in Figure 6a for Asus OLED, 10-bit, that in the upper half, the summed values do not align perfectly with the grayscale luminance values as is the case for the rest. However, after looking into the statistics of this deviation, it showed that the maximum this deviation reaches is ⩽2%, which should not raise any major concerns.

**Table 2.** Additivity test checks whether the maximum luminance of $R_{max}+G_{max}+B_{max}\overset{?}{=}White$.

Display/luminance (cd/m^2^)	R+G+B	Max white
Asus OLED	254.3	252.7
Samsung OLED TV	192.7	192.1
LG UltraGear	249.1	247.1
PROPixx	128.1	128.1

### Linearity and gamma function (γ)

After setting everything to user-mode/manual, adjusting to the aforementioned settings, and running color calibration, we found the following gamma values and summarized them in Table 3 for the different displays. All displays were calibrated for γ = 2.2; however, upon fitting a power function into their various output luminance curves, a deviation in the encoded gamma values has come to light and was more noticeable in certain displays than in others. For instance, Samsung OLED TV shows the least deviation of its gamma values across its RGB channels and the grayscale (⩽0.01). In contrast, Asus OLED display shows a small yet noticeable difference between its red-channel gamma value and the rest of the channels, which amounts to a maximum difference of approximately 0.1. The LG LCD display gamma values across its channels show a deviation of no more than 0.09 at most between the green channel and the blue channel. PROPixx, as it was mentioned before, comes with perfect linear behavior that governs all its channels combined or individually.

**Table 3.** Gamma curve fitting of RGB channels and grayscale of each tested display after color calibration.

Display/gamma (γ)	R	G	B	Grayscale
Asus OLED	2.24	2.17	2.14	2.18
Samsung OLED TV	2.30	2.29	2.29	2.29
LG UltraGear	2.04	2.07	1.98	2.01
PROPixx	1.0	1.0	1.0	1.0

### Color gamut

**Figure 7.**
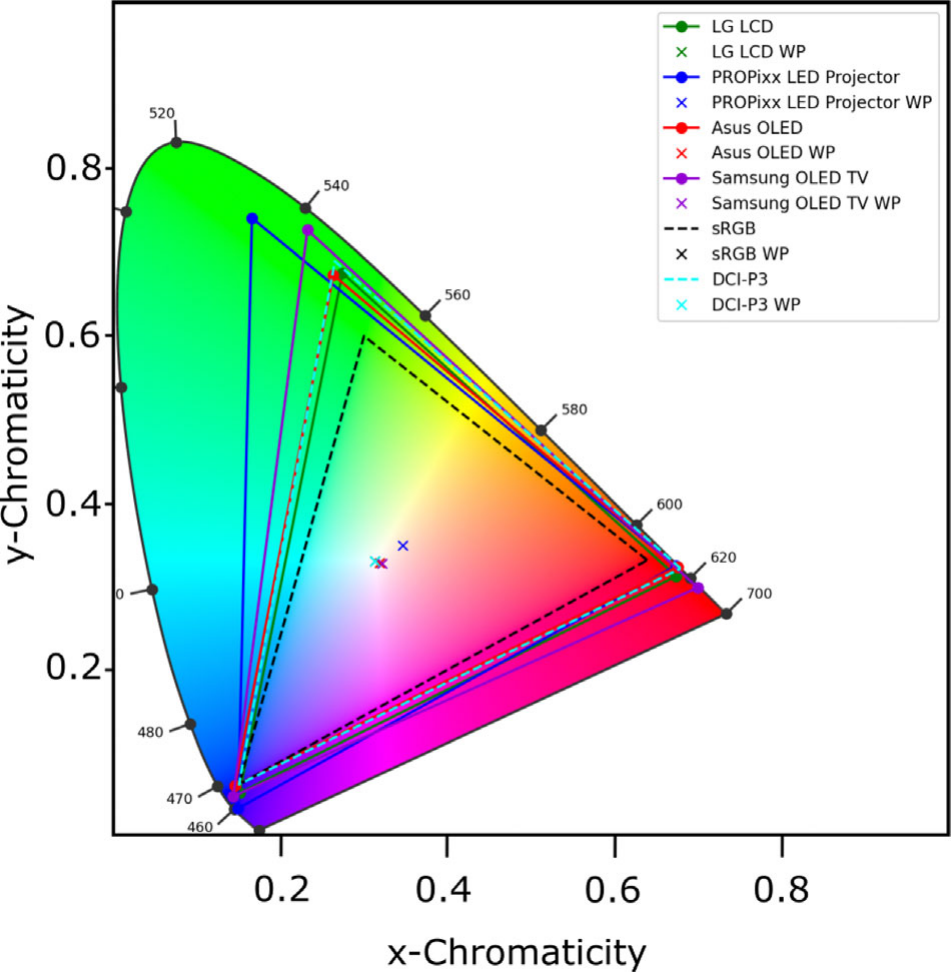
Color gamut of the four tested display systems represented on CIE 1931 xy-Chromaticity diagram with their corresponding white-points (WP). sRGB and DCI-P3 color gamut are plotted as references. Note that the PROPixx in High Bit-Depth mode has not been calibrated to D65, unlike the other monitors.

A representation of each color gamut can be seen in Figure 7 along their WP; precise chromaticities are provided in Table 4. In the Figure 7 the common small *sRGB* and the wider *DCI-P3* color spaces are represented in dashed lines as references. Both Asus OLED and LG LCD approximate very closely the DCI-P3 color gamut, whereas Samsung OLED shows a larger color gamut thanks to its quantum-dots technology and the narrow (pure) primaries it can achieve. PROPixx also shows yet a larger color gamut, hence a richer color experience, thanks to the optimized selection of its RGB LED primaries. All WP seem to align with the D65 WP, except for PROPixx which seems to be a little bit warmer and lies around D50. This behavior likely has two sources: first, we were not able to calibrate the color profile of the PROPixx as for other monitors using our calibration software. Second, our measurements are made in the PROPixx’s “High Bit-Depth” mode, which results in slightly cooler LEDs than the default RGB 120-Hz mode and therefore a shifted WP (Peter April, personal communication). One would be able to calibrate a D65 WP for the PROPixx in High Bit-Depth mode using a custom LUT if desired.

**Table 4.** xy-Chromaticity coordinates of displays’ primaries RGB and white-point (WP). Note that the PROPixx in High Bit-Depth mode has not been calibrated to D65, unlike the other monitors.

	Red		Green		Blue		White-point	
Display/xy-chromaticity	x	y	x	y	x	y	x	y
Asus OLED	0.676	0.322	0.263	0.673	0.145	0.0582	0.319	0.327
Samsung OLED TV	0.700	0.297	0.232	0.727	0.143	0.045	0.319	0.323
LG UltraGear	0.675	0.311	0.274	0.676	0.151	0.050	0.319	0.327
PROPixx	0.674	0.323	0.166	0.742	0.149	0.031	0.346	0.348

### Luminance uniformity

**Figure 8.**
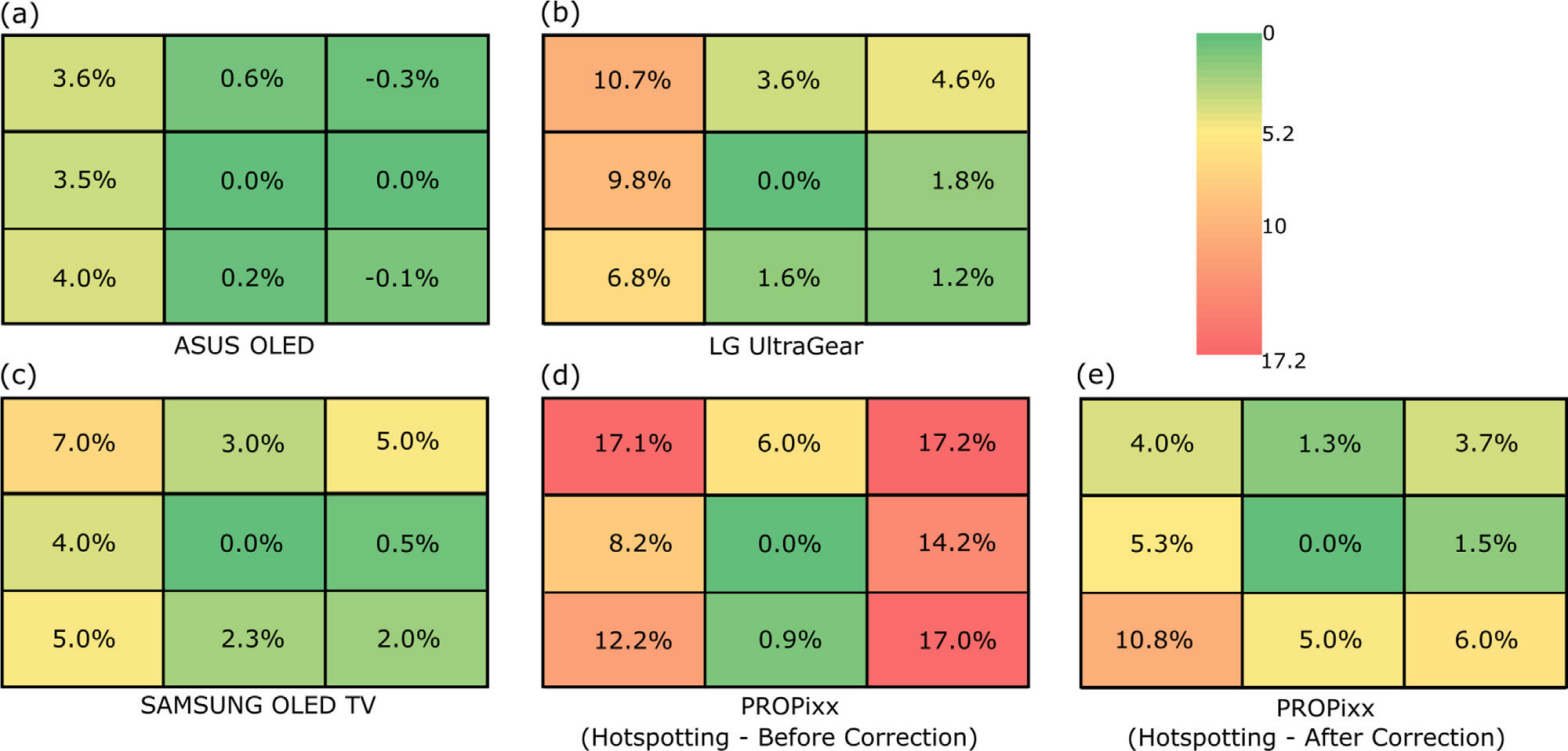
Luminance uniformity across each of the tested displays. The display is divided into 3 × 3 grid and luminance was measured at the center of each. The difference is reported as an average percentage relative to the central part of each display. Negative values indicate that the luminance level at that part of the display is higher than the central part. The heatmap is universal across all the displays and in absolute value.

Luminance uniformity across a display ensures that presenting a stimulus with a fixed luminance level at different parts of the display does not result in a dimmer or brighter representation depending on its location. We divided the display into a 3 × 3 grid (i.e., 9 sections) and measured the luminance ramp of a grayscale in each section individually. Figure 8 shows the average difference between the eight sections of each display relative to its central part, which is usually considered to be the most stable part of any display system. The negative numbers indicate that higher luminance levels of that part of the display than the central part were registered.

The LG LCD display shows a higher average difference than the other two displays Asus and Samsung OLED across its surface with a difference as high as 10.7% in the worst-case scenario. It seems that the left half of the LG LCD display performs far worse than the right half. Both OLEDs have maximum differences that amount to approximately 4% and approximately 7% for Asus and Samsung, respectively. The PROPixx projector suffers from what is known as *hotspotting*, which is a well-known problem in projectors and causes strong variations in luminance across the projection area, in our case up to 17.1% difference. VPixx Technologies offers a calibration procedure in which the effect of hot-spotting can be mitigated. Figure 8e shows the result of luminance difference after running hotspotting correction in which the luminance uniformity across the display has improved. However, the left-bottom corner still shows a maximum luminance difference as great as 10.8%. It is worth mentioning that after the correction, the maximum luminance level measured at the center dropped from approximately 128 to 97 cd/m^2^. Out of the four tested displays, Asus OLED seems to show the most luminance uniformity across its surface.

### Filling factor

We measured the luminance output of a rectangular stimulus presented in the middle of the display starting at dimensions of 10% of the display resolution and going up to full-screen 100% with an increment of 10%. The background color was set to one of three options: black, white, or mid-gray. The stimulus color was either one of the primaries (R, G, or B) or grayscale (black to white), and depending on the available bit depth ranging from either [0–1023] for 10-bit displays with a step of 50 or [0–4095] for the 12-bit PROPixx with a step of 100.

**Figure 9.**
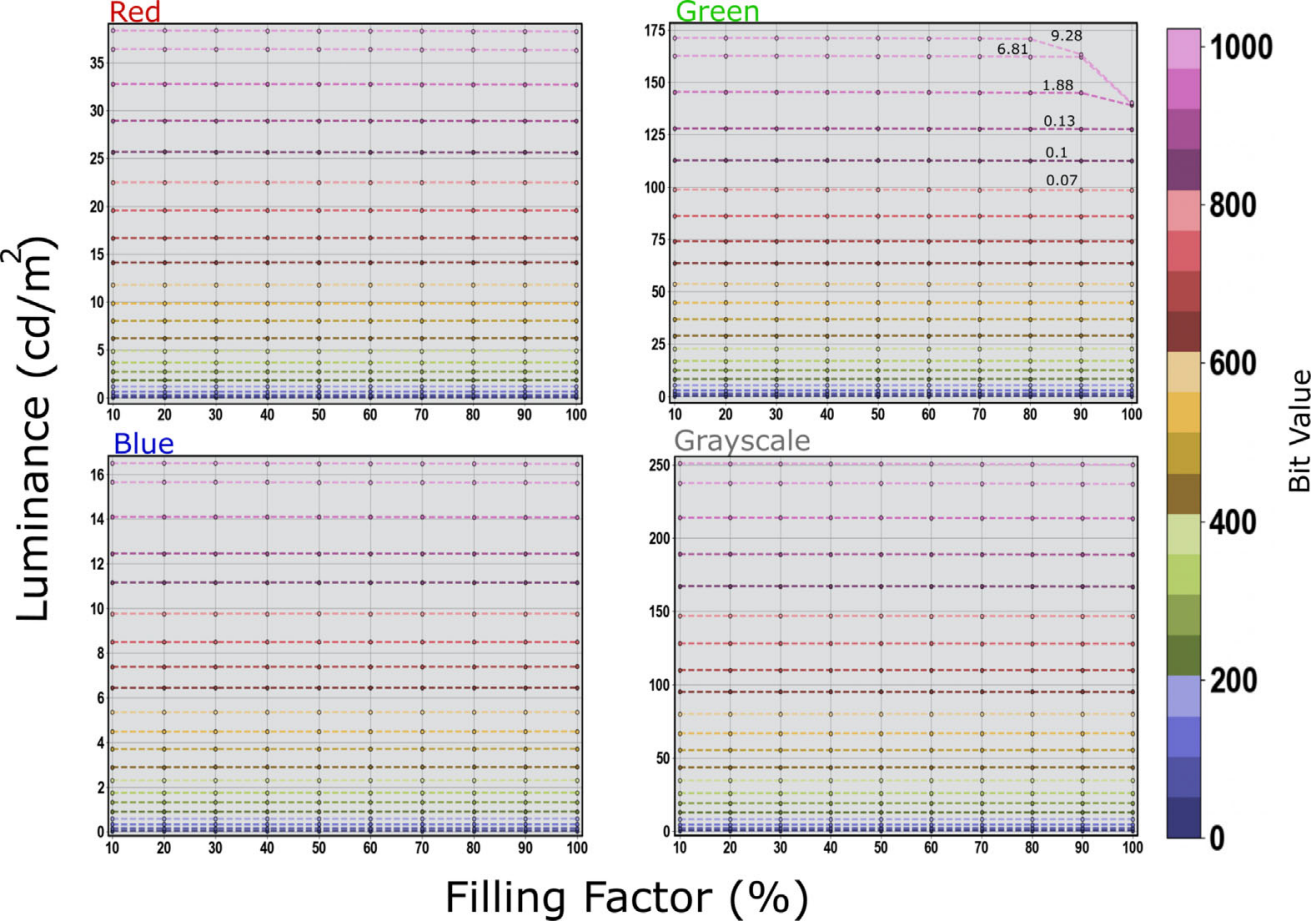
Luminance output as a function of filling factor (percentage of the stimulus area) for different bit values [0–1023] with a step of 50. The filling factor influence (aka. APL) is tested on Red, Green, Blue channels and the grayscale (black to white). The background color is set to mid-gray. All the channels show steady luminance output independent of the filling factor except for the upper range of the bit values, greater than 900, of the green channel where it shows a standard deviation up to 9.28 and a drop in luminance from approximately 171.3 to approximately 140.3 cd/m^2^. A few standard deviation values are written on top of tested bit values.

**Figure 10.**
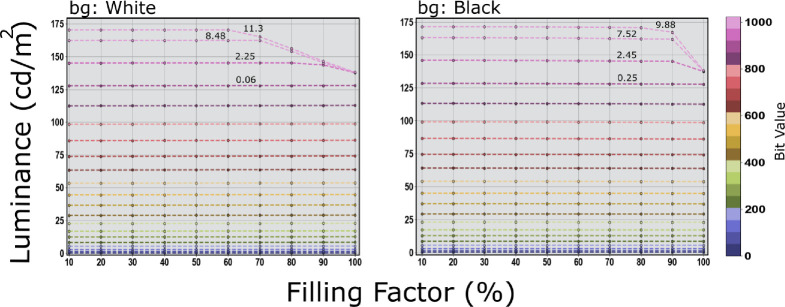
The effect of the background color on the luminance output of the Asus OLED Green channel. A white background (left) affects the luminance output at higher bit values causing a standard deviation across the various filling factors of 11.3 and luminance change from approximately 170 to approximately 138 cd/m^2^ starting from a filling factor 60% up to 100%. The black background (right) shows a maximum standard deviation of 9.88 and luminance change from approximately 171.5 to approximately 137.9 cd/m^2^.

The measurements for the three display systems, Samsung OLED TV, LG LCD UltraGear, and PROPixx projector, did not show any luminance dependency on the filling factor or the background color. The maximum reported standard deviations across all the measurements of R, G, B, and grayscale and different filling factors were found to be 0.97, 0.12, 0.94 for the Samsung, LG, and PROPixx, respectively when the background is set to mid-gray. Changing the background color did not show any sign of larger deviation or abnormal luminance fluctuation worth the attention. For the Asus OLED, however, a deviation in the luminance behavior was observed only for the green channel and only at a very high bit value >900 in 10-bit depth. A drop in luminance starts to kick in after a filling factor of greater than 90% and after 80% for a bit value of greater than 950 and of greater than 1000, respectively when the background is either black or mid-gray, check Figure 9 (green) and Figure 10 (bg:black). When the background is rather white (all the background pixels are at their maximum) a drop in luminance starts to be noticeable at a bit value of 950 or greater and a filling factor of greater than 70%, and as early as a filling factor of 60% for the maximum bit value 1023, check Figure 10 (bg:white). The standard deviations across different filling factors for the green channels with mid-gray background, specifically for the last six tested values [800, 850, 900, 950, 1000, and 1023] are [0.07, 0.1, 0.13, 1.88, 6.81, 9.28] respectively as can be read on the green channel plot Figure 9 (green). Only a few standard deviation values are written on top of the last four lines that correspond with the tested values for the green channel with different background as they are the most affected values out of all the other measurements. The maximum standard deviation of 9.28 is the result of a maximum drop in luminance under the testing settings from approximately 171.3 to approximately 140.3 cd/m^2^. When changing the background color, only the green channel kept showing a drop in luminance and only for bit values of greater than 900. For the white background (all the background pixels at their maximum) the standard deviation reaches 11.3 for the maximum bit value 1023 and shows a drop in luminance from approximately 170 to approximately 138 cd/m^2^ across different filling factors. Surprisingly, when the background is set to black (all background pixels are off) the maximum standard deviation is still comparable with when the background is set to mid-gray and amounts to 9.88 and the change in luminance across the various filling factors goes from approximately 171.5 to approximately 137.9 cd/m^2^. We refrain from plotting the rest of the measurements for the other display systems because of the little deviations they show as they seem to keep their luminance output behavior steadier and constant regardless of the filling factor and the background color.

### Pixel response time and waveform

**Figure 11.**
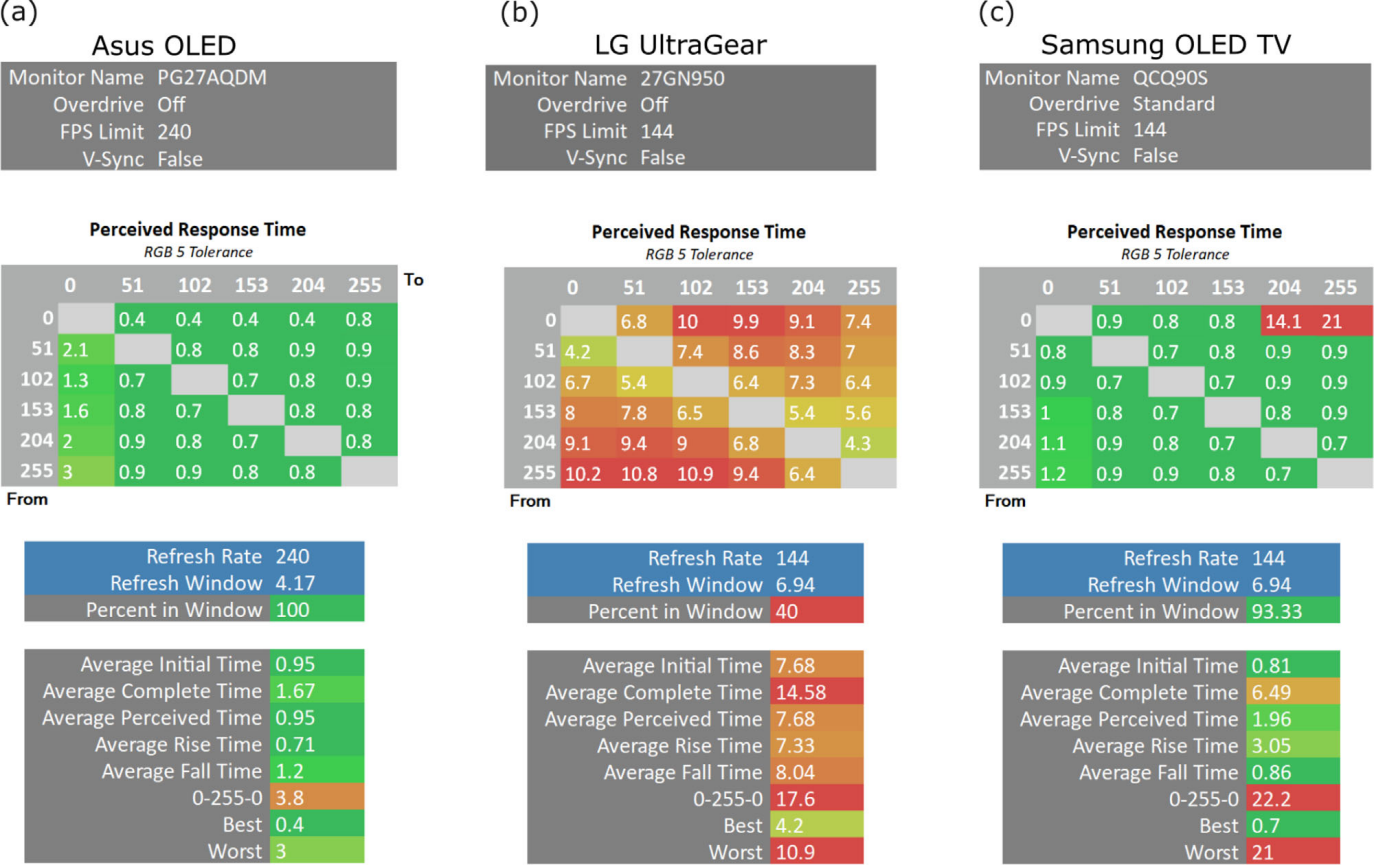
The transition time of pixel responses (Gray-to-Gray; G2G) between six uniformally spaced gray levels reported in milliseconds with a tolerance level of 5 RGB values for the 3 display systems Asus and Samsung OLED, and the LG UltraGear LCD using OSRTT PRO CS.

Pixel response is how long it takes a pixel or a group of pixels to make a transition from one state to another to output different colors or light levels. The most extreme transition would happen when the two-pixel values are very far apart i.e., black to white or vice versa. OSRTT response box allows us to measure how long it takes on average for a full-screen color to make a transition in what is known as Gray-to-Gray transition i.e., All pixel transitions happen in the grayscale range [0–255] after scaling the actual bit depth of the selected display down from, for example, 10-bit to 8-bit. The step between every two consecutive gray colors is uniform and calculated by dividing the whole range into six parts, that is, a step is 255/5 = 51 plus the black (0). Figure 11 shows the response time in milliseconds for the three display systems, a) Asus OLED, b) LG LCD, and c) Samsung OLED TV, each with their corresponding native refresh rate as indicated under *“Refresh Rate”*. Each set of measurements contains four subtables, top to bottom they are: 1) The table on the top contains information about the display model under the test, whether overdrive is activated, the maximum tested frames per second limit, and whether V-Sync is activated if available. 2) The second table contains the transition time in milliseconds between every pair of gray colors (G2G) expressed as *“Perceived Response Time”*. 3) The third table contains information about the detected settings of the display such as the refresh rate, the actual time of the refresh window in milliseconds, and the test window size (100 for full-screen). Finally, 4) the table at the bottom contains summary information about the measurements regarding the average initial time, which is the metric recommended for measuring the response time—that is between 10% and 90% of the signal ignoring any over- or undershoot if any occurred (International Committee for Display Metrology, 2023a). The average complete time, that is, until the signal reaches effectively the target value and stabilizes. The average perceived time, that is measuring the signal until it is within the predefined tolerance including any over- or undershoot if it happens. Then the average rise time and average fall time express the rising and the falling time of the signal from one state to another in milliseconds. 0-255-0 is the total transition from black to white and back to black. Best and worst are self-explanatory for the best and the worst transition time in this set of measurements.

Perceived response time differs from the initial response time measurements because it keeps measuring the signal up until after any overshoot or undershoot if either was part of the signal. In other words, until it settles close to the end target state within a predefined tolerance; here, the tolerance is set to 5 RGB bit values. The smaller the transition time the more control the display has over its pixel states, hence offering more readiness for an accurate change on-demand with little to no delay. We focus on the perceived response time in this report because it is more realistic and relatable to the human experience in vision science experiments.

Looking at the measurement of the LG LCD in Figure 11b shows the well-known issue of LCD IPS that requires on average 7.68 ms to make a change to the liquid crystal from one state to another, in line with other findings (e.g., Elze & Tanner, 2012). Whereas Asus OLED shows a very fast response time (Figure 11a), with an average of 0.95 ms and worst is 3 ms, which is still below the time required for displaying 1 frame at its maximum refresh rate 1000 ms 240 Hz =4.16¯. On the other hand, Samsung OLED TV shows an average transition time of 1.96 ms and the worst is 21 ms for the transitions toward white 255. While its signal fall-time looks very reasonable for an OLED display, which is 0.86 ms, its rise-time on the other hand looks a bit sluggish with 3.05 ms on average when compared to Asus with 1.2 ms and 0.71 ms respectively. At the time of writing the manuscript, we could not justify why only the transition to the gray level 204, 255 in Samsung OLED TV accounts for too much delay in the signal in comparison to other gray levels and to the Asus OLED display in general.

For PROPixx, this test was not possible to run because the OSRTT software could not communicate with the projector setup. Hence, the corresponding data could not be measured.

#### Waveform

We recorded measurements of a single frame during a transition from black to white to black (0-255-0) in three ways. First, with a static flashing stimulus (i.e., a 512 × 512 white box in the middle of the display in full-screen mode with a mid-gray background) for exactly one frame every second for at least 10 seconds. Second, the same flashing stimulus but this time displaying it for a period of 11 frames every second. Finally, the same stimulus translated across the display from left to right at a speed of 50 pixels per frame. Doing so allows us to measure closely the time it takes to process one frame, to see if that is influenced by a longer presentation of a stimulus or a moving stimulus, and to measure the signal’s waveform more closely. This part of the experiment was coded in *Psychopy*, with careful attention paid to minimizing and removing dropped frames. We used OSRTT response box hardware and software to record the signal using its live mode.

**Figure 12.**
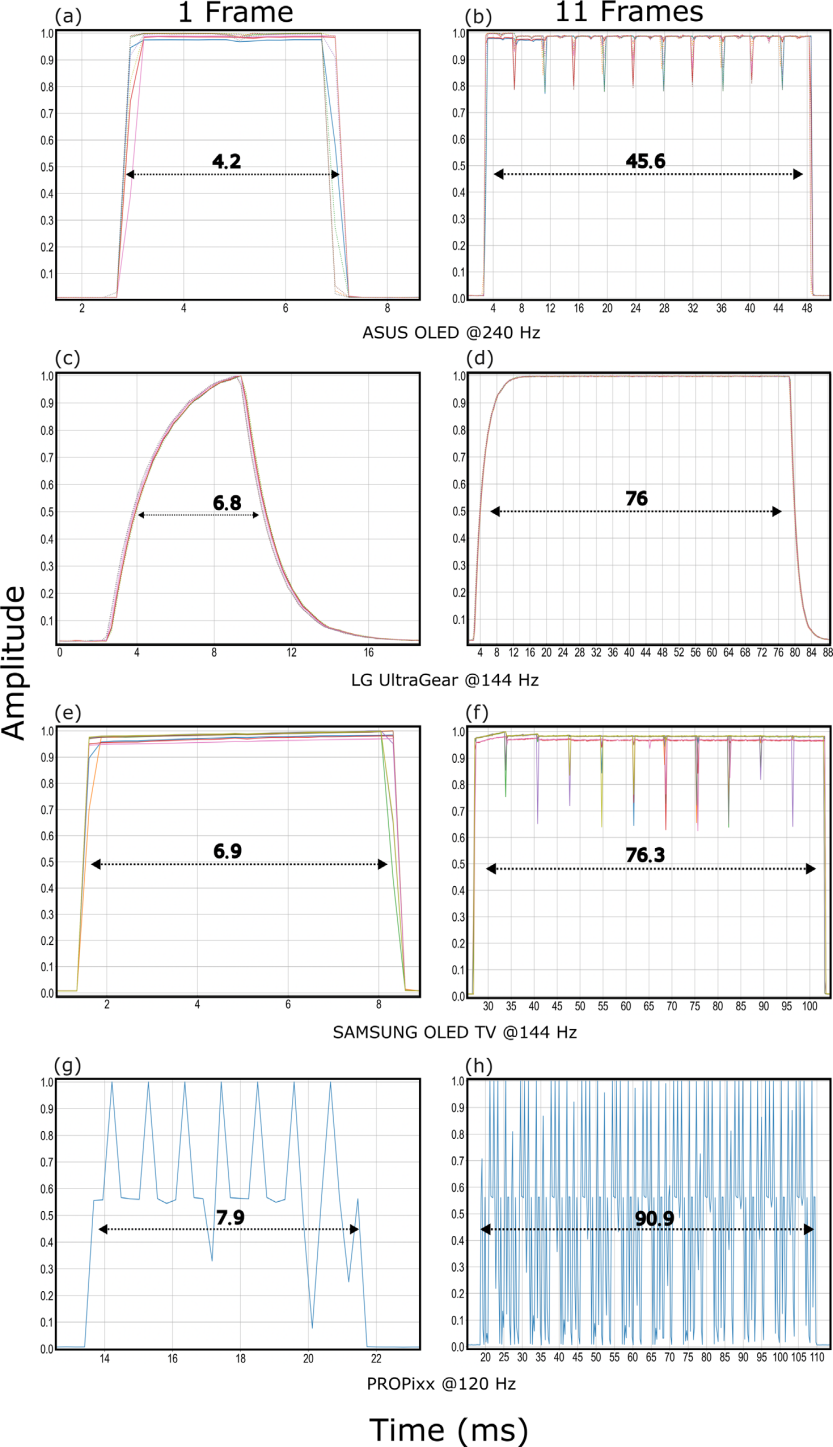
Waveform measurements for 1 frame and 11 frames a second at the native refresh rate of each corresponding display system. Nine measurements in total superimposed on top of one another for each except for PROPixx measurements where this was avoided owing to the jittery waveform that would make the figure very cluttered otherwise. The unit of the measurements is milliseconds, an average approximation of the 9 measurements is written on each subfigure. Note that the x-axis scale differs across subfigures.

Figure 12 shows the waveforms of pixel responses for 1 and 11 frames for the 4 display systems and the average duration it takes for this transition to happen averaged across 9 measurements in total for each. The durations are calculated at the signal’s FWHM, that is, at 50%. As expected, the plots show a sharp rise and fall of the signal for the OLED and PROPixx DLP projector with a very accurate duration when presenting the specified stimulus. E.g. Samsung OLED TV can be driven at a maximum refresh rate of 144 Hz a second, hence 1 frame should last 1000 ms 144 Hz =6.94¯ ms → 11 frames would last approximately $76.3\bar{8}\;\mathrm{ms}$. Even though the LG LCD shows an accurate duration of presenting the stimulus for a certain number of frames at its FWHM, it is clear from its waveform that the presented stimulus reaches its desired luminance level (1.0) only for a brief period for the liquid crystal requires a considerable amount of time to change its state which is evident in the signal rising and falling shapes. In contrast, the OLED and DLP technologies show instantaneous rise and fall signal response, hence the stimulus is presented for exactly all the duration of a complete frame at its desirable luminance level. The DLP PROPixx projector waveform looks very spiky compared to all other systems owing to how the micromirrors in DLP systems operate.^1^

**Figure 13.**
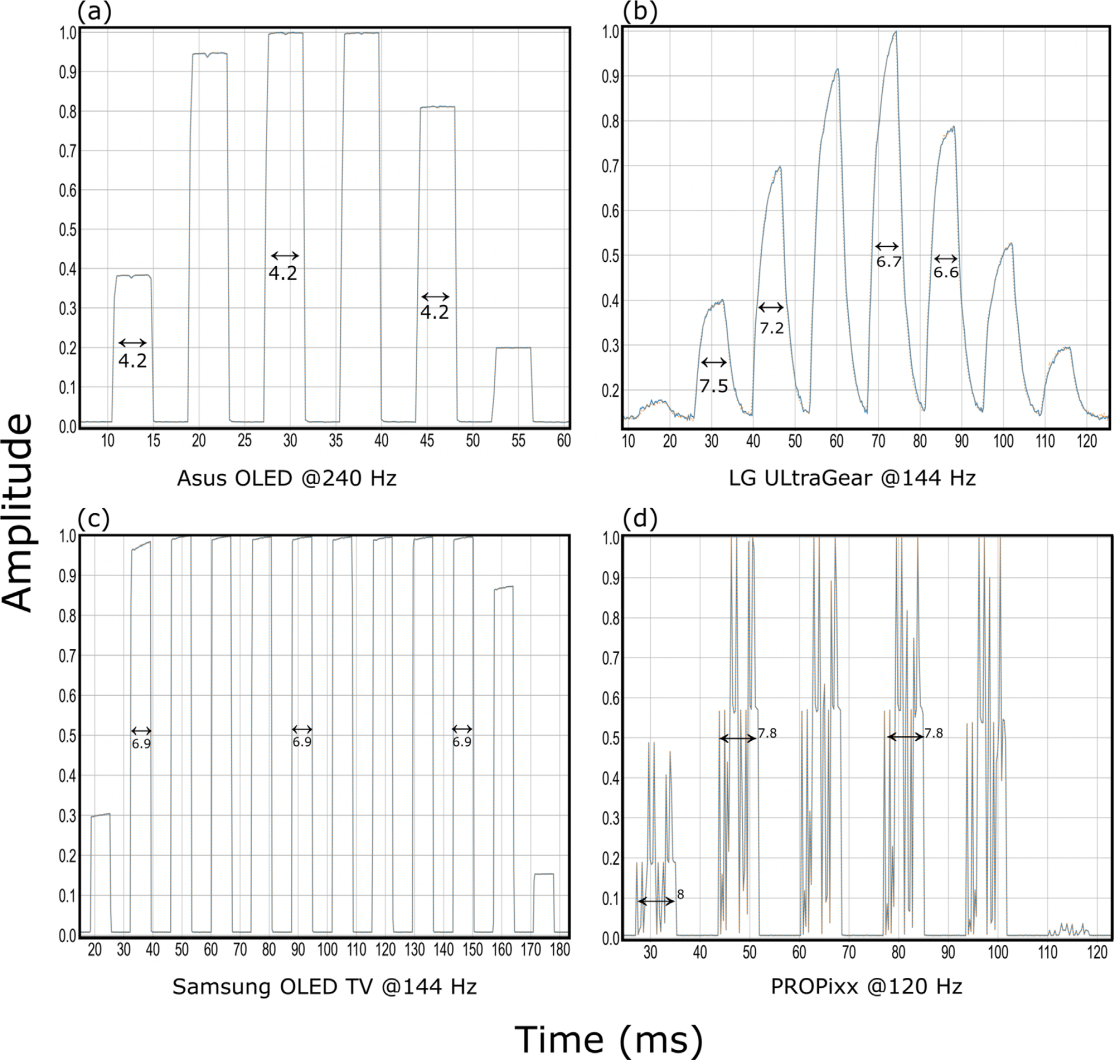
Measuring a moving stimulus (rectangle) horizontally from left to right across each display. The photodiode sensors are also placed horizontally, hence the waveform response’s progressive growing/diminishing amplitude. Note that the x-axis differs between subfigures.

For a moving stimulus, the response box (photodiodes) was placed horizontally while the stimulus moved left to right several times. Figure 13 shows the response signal as the stimulus moves across the sensors, hence the progressive growing and diminishing of the amplitude. The average signal duration is given on each subplot accordingly given the refresh rate the display was operating at. For both OLED displays, there is no observable change in the signal response behavior, while for LG LCD the response varies while the stimulus moves across the sensors ranging from 6.6 to 7.5 ms at 144 Hz. PROPixx projector display shows a little deviation from its theoretical value of 1000 ms 120Hz=8.3¯ ms  that amounts to a maximum of 0.5 ms.

## Discussion

The metrics we used in this report to assess the quality and suitability of different displaying systems show that it is hard for a consumer-level display system to meet all the stringent requirements a vision scientist may demand for various experiments. Even within the same model, variations and inconsistencies are unsurprising, since the primary focus of consumer-level displays is not precise and accurate physical display, but rather for daily activities such as movie watching, gaming, and similar tasks. Consumer-level display technology, nonetheless, has come a long way since CRT displays became the vision science gold standard. Here, we have tested four of the recent and most advanced displaying systems to evaluate their suitability for vision science. Three systems are consumer level and relatively budget friendly, while the other system is considered a professional scientific device, namely the PROPixx projector.

Testing the behaviors of the luminance curves and their encoded gammas, after careful selection of settings for each display and after color calibration, showed that all three consumer-level displays (Asus OLED, Samsung OLED TV, and LG LCD) follow a predictable gamma power function that enables an experimenter to decode the gamma and linearize their output if desired. The gamma values of each of the RGB channels and the grayscale luminance curves are presented in Table 3 and can be seen visually in Figure 3. As Cooper et al. (2014) concluded in their paper, it is possible to have luminance output following a power law (gamma function) upon careful settings selection so that the monitor output is linearizable. Our color calibration process did not affect the luminance monotonic ramps, at least not for the small-patch mode, rendering the whole bit depth to be useable unlike the findings in Ashraf et al. (2024) paper where after performing color calibration ceiling/saturation starts to kick in around a bit value of approximately 700 in a 10-bit depth in the small-patch condition.

The measured area for each display is a 512 × 512 in the center while the background is set to mid-gray unless otherwise is mentioned. During our tests, we did not notice any difference in the luminance behavior if the background changed from mid-gray to black. However, changing the color patch size from 512 × 512 to full-screen patch size resulted in a change in the luminance behavior only for the Asus OLED display system and only for the red and green channels, where these channels started to saturated at bit values of approximately 849 and 916, respectively, in a 10-bit depth range. Whereas for all the other displaying systems, no change in their luminance behavior has been recorded, showing an improvement in hardware behavior compared to the tested hardware in Elze et al.’s (2013) study, in which saturation in full-screen mode was an inevitable characteristic for their tested hardware.

In another test, we checked if maxing up two of the three RGB primaries would influence the behavior of the third primary by saturating two channels and ramping up the remaining one. We chose to fix two channels at the maximum luminance level, rather than at any lower value, based on the hypothesis that the firmware’s behavior would be most noticeable when regulating the subpixel outputs at their highest luminance. At this level, heat generation becomes a significant issue, and mitigating it would likely take higher priority for the firmware to take care of than achieving the requested luminance. The results are plotted in Figure 5. In the case of Asus OLED and because it relies on four-subpixel primaries, RGBW, one notices that the blue channels, when the red and the green are saturated, start to exceed the maximum recorded luminance level that of the calibration, and exceeds the maximum measured luminance of its grayscale ramp. We understand this behavior in the light of Chesterman et al. (2016)’s study that pointed out that in RGBW systems usually representing white color activates not only the white subpixel nor only the combined maximum of the three RGB primaries, but rather a combination between the white subpixels and other subpixels as well. Given that, we suspect that while fixing the red and the green channels to the maximum and ramping up the blue channel to reach the white color, along the bit range the white subpixel started to be active and act together with the changing subpixel, hence the overreaching in the luminance level. Samsung OLED, in contrast, did not show a similar behavior owing to its use of a different technology (Quantum-Dot).

In the additivity test, all four display systems showed a very nice behavior in which their individual RGB channels summed up to the corresponding luminance level when they act combined to reproduce grayscales. The results were summarized in Table 2 and visualized in Figure 6. These findings are consistent with the measurements of Cooper et al. (2014) and Ito et al. (2013), in which their measured OLEDs showed a nice additivity behavior with a maximum difference in luminance levels between (*R*_*max*_ + *G*_*max*_ + *B*_*max*_) and *white* to be less than 1.0%.

Measurement of the color gamut across the four display systems revealed that the PROPixx, followed by the Samsung OLED TV, exhibited the widest color gamut (Figure 2). In comparison, the Asus OLED and LG LCD systems covered up to approximately 94% of the DCI-P3 color space. A larger color gamut allows a richer color experience, more saturated colors, and more color variations. The color gamut is a result of the selection of the underlying primaries, the narrower their spectral curves the larger the color gamut they define. All the displays’ color gamut are larger than the standard *sRGB* and some even larger than *Adobe RGB 1998*, making them very suitable options for experiments requiring a wide range of colors.

The luminance uniformity test showed that Asus OLED has the least luminance variation across its display surface (Figure 8a), when dividing the display into a 3 × 3 grid, measuring grayscales and comparing them against the central part of the display/grid. All three consumer-level display systems show worse average differences on the left half of the display (Figures 8a–c), which we assume is due to the electronics concentration on that side. The PROPixx projector suffers from a phenomenon called *hotspotting* in which it exhibits a highly non-uniform luminance behavior across its display surface. After calibrating and correcting for hotspotting, the variation in luminance improved from a maximum average difference of 17.1% (before) → 10.8% (after). The left-bottom corner of the projector display did not show much improvement before and after the correction in comparison to the other parts of the grid (Figures 8d vs. 8e). It is important to remember that the properties of PROPixx are a function of the projected image size and distance, that is, the hotspotting effect, among other characteristics, would differ depending on these parameters. To the best of our knowledge, there are only a few luminance uniformity measurements for OLED displays in the literature to this date. Ito et al. (2013), for example, measured the uniformity of Sony PVM-2541 OLED, the same model used in the Cooper et al. (2014) study and found little to no difference in luminance output across the display surface when measured from a distance and along the surface normal, the maximum reported difference is less than 3%. Our measurements show a difference of maximum approximately 7% for the Samsung OLED TV and maximum approximately 4% for the Asus OLED gaming monitor given that the displays are color-calibrated.

The measurements of the APL or as mentioned here the filling factor show no sign of abnormal or sudden change in luminance output that could depend on the stimulus size or the background color for the three displaying systems (Samsung OLED TV, LG LCD UltraGear, and the PROPixx projector). All these three systems were invariant to the filling factor (stimulus size) and the background color (black, white, or mid-gray) with a maximum standard deviation of 0.97, 0.12, and 0.94 for the Samsung, LG, and PROPixx, respectively when the background is set to mid-gray. However, only the green channel of Asus OLED gaming display shows a sign of unsteady luminance output, under the selected test conditions, and only for bit values of greater than 900 in a 10-bit range and a filling factor of more than 60%. The Asus green channel suffers the most when the background is set to white (all background pixels at their maximum), causing a maximum standard deviation of 11.3 caused by the change in luminance across the various filling factors, starting from filling factor of greater than 60, from approximately 170 to approximately 138 cd/m^2^, check Figure 10 (bg:white). The black and the mid-gray background both show standard deviation values of 9.88 and 9.28, respectively. Surprisingly, even when all the background pixels are off (black), this does not seem to help regulate the luminance output of the green channel better. Notably, the common way of testing APL is by targeting the grayscale range (black to white) regardless of the behavior of the individual RGB channels. Our APL grayscale measurements show very robust behavior in luminance output regardless of the background color (black, white, or mid-gray) with a maximum standard deviation of 0.27. The unexpected luminance change happening in the green channel should not be a practical limitation but more of a technical issue under certain conditions. As long as the green channel is not being used under extreme conditions, for example, filling factor of greater than 60% and bit value of greater than 900, this should not impose any practical limitation on the display performance. These findings show an immense improvement from the reported results in previous reports such in Ito et al. (2013); Tian et al. (2019); Yang et al. (2022).

Regarding the pixel response time, consistent with earlier reports (Cooper et al., 2014; Elze & Tanner, 2012; Ghodrati et al., 2015), we find that the tested LCD display exhibits sluggish behavior (Figure 11b). In contrast, both OLED devices show a very quick response, as little as 0.3, 0.4 ms (Figures 11a and 11c). Ito et al.'s (2013) analysis suggests a similar or slightly slower transitional duration of approximately 2 ms to reach a target luminance level. However, the Samsung OLED TV performs poorly for the transition between gray values of 204, 255, with sluggish response that goes up to 21 ms at 144 Hz, which is far beyond the theoretical duration of a single frame 1000 ms 144 Hz =6.94¯. During the time of writing the manuscript, it remained unclear to us why this behavior keeps repeating only for a transition towards gray values of 204, 255. With this exception, based on our measurements it seems that OLED response time is still fast enough and accurate for, virtually, any perception experiment.

The Asus OLED proved to be very accurate and precise in its response time behavior, for its slowest response would take only 3 ms, which is still under its theoretical frame duration 1000 ms 240 Hz =4.16¯ (Figure 11a). Analyzing closely the waveform of the pixel response (Figure 12) shows the instantaneous and sharp rise and fall of OLED signal for Asus and Samsung OLED, which enables presenting the desired stimulus at its target luminance level for virtually the complete period of one or more frames as requested. On the other hand, by observing the waveform of LG LCD pixel response, one sees how presenting a stimulus for exactly 1 frame would not faithfully present it at its desired luminance level for the whole frame duration but rather only for a brief period, being affected by the gradual rise and fall of the signal as a result of turning the liquid crystals that eat up a considerable amount of time of the total frame duration (Figure 12c; see also Elze & Tanner, 2012). The PROPixx waveform is also sharp and square; however, the spikes one can observe in the recorded data is due to the photodiode interacting with the DLP flipping micro-mirror inside the projector. These spikes should not have any effect on the target luminance level. While capturing a moving stimulus that moves from left to right across the display, the Asus OLED, Samsung OLED TV and PROPixx projector, all have sharp, clear, and instantaneous signal (Figure 13). In contrast, the LG LCD shows a variation in the presented signals of the moving stimulus for it varies between 6.6 and 7.5 ms for a stimulus that is supposed to have a duration of only 1000 ms 144 Hz =6.94¯ ms  (Figure 13b).

Contemporaneous results to ours have recently been published as a preprint by Dimigen and Stein (2024). Specifically, they evaluated the same ASUS ROG Swift OLED PG27AQDM that we do in this paper, and compared this to two LCD monitors (an ASUS and an Iiyama) and an Iiyama CRT monitor. Compared with our measurements, their paper focused more on the temporal properties of monochromatic stimuli. They included a number of measurements that we did not make, including dependence on viewing angle and warmup time (temperature). In addition, they included a test of high-frequency flicker stimulation and an intra-saccadic stimulation experiment. In contrast, our paper more thoroughly characterizes the long-presentation luminance and color properties of this monitor.

Similar to our measurements, they find that the ASUS OLED spatial luminance uniformity does not exceed 5.5% in any two locations they measured across the display surface (grid of 3 × 5). Even though they followed a different technique to ours, the spatial uniformity deviation is very close to one another (*max* 5.5 vs. *max* 4.0 %). The transition times they recorded for the signal rise and fall are approximately 0.3 ms. Our evaluation of the signal rise and fall time were slightly different with approximately 0.7 and 1.2 ms, respectively. Nonetheless, the FWHM measurements of the signal duration of one frame align very well in both papers with one another conforming with the expected nominal value at 240 Hz. Dimigen and Stein (2024) show how activating “Uniform Brightness” helps in having more controlled ABL behavior and limiting the occurrence of dimming depending on the display content. However, they find that this option is only of use under Windows OS, while testing it on Linux OS showed some hardware intervention changing the luminance output when display brightness is set to 100%. They advise, hence, when using Linux OS to limit brightness to only 40% (approximately 140 cd/m^2^). Our results instead suggest that under Linux OS it is still possible to make use of the “Uniform Brightness” option. We did not see this drastic drop in luminance in relation to the filling factor (APL) when the display brightness is still 80% (calibrated for approximately 250 cd/m^2^) when testing the whole range of a grayscale (black to white). Nonetheless, our APL measurements for the grayscale still align with Dimigen and Stein (2024) by showing a robust luminance output independent of the APL percentage or the background color (black, white, or mid-gray). In general, the similarity of these results from two different laboratories using different control hardware and measurement equipment should instill confidence in the reader that the overall good performance of the ASUS OLED we report here is unlikely to be accidental.

### Caveats and limitations

It is important to note that the measurements and findings we present are specific to the tested display units under the selected settings and calibration methods. As a result, similar performance across identical models cannot be assumed or guaranteed. Informally, we observe that the behavior of the displays is significantly influenced by the chosen settings. Although we took significant effort to select settings that produced the best possible performance for each monitor over a variety of stimulus aspects, we cannot guarantee that better (or worse) performance could not be found, nor can we guarantee that different exemplars of the same display devices will perform similarly.

All our luminance measurements were taken after running color calibration on the corresponding systems, except for PROPixx, which would affect the luminance output and the behavior of the RGB primaries. To our knowledge, all related literature relies rather on the default display profile while only tweaking the preset options for color spaces and gamma by dialing it directly on the display hardware. In our experience, the hardware preset and the default display profile would not reflect faithfully and accurately the selected values and can suffer from inaccuracies. We advise performing color calibration and profiling so that the display profile is manually and accurately tuned to the desired peak luminance, and the behavior of the display primaries is appropriately corrected for the desired gamma and luminance output, besides having accurate color reproduction if one desires. One has, as well, the option to calibrate only for luminance output, that is, grayscale, and omitting color accuracy if color is not of any importance to the target experiment.

## Conclusions

We find that at least two consumer-level OLED display devices may be suitable for vision science experiments (holding important caveats for each). The Asus OLED ROG Swift PG27AQDM performed very well across our test battery, with the exception that its red and green channels saturate in full-screen mode measurements, and the green channel showed changes in luminance output as a function of stimulus size at high intensities. The Samsung OLED TV also performed very well, except for the pixel response time test, in which it performed very poorly for only certain G2G transitions. The professional PROPixx projector system performed very well in all tests, except for spatial uniformity due to hotspotting, but remains the best over all monitors we tested (albeit with a price tag some 20 times higher than the Asus). In summary, consumer-level display systems seem to have improved since previous reports and offer opportunities for vision scientists to use high-performance systems for relatively little cost compared to professional displays.

## Footnotes

1 A curious reader may consult (Lee, 2008) for how digital micromirror devices work.

## Appendix: Monitor settings

This appendix describes the settings used for each of the consumer monitors.

For the ASUS OLED display we have adjusted the display settings to the following: Brightness: 96, Contrast: 80, Uniform Brightness: On, Color Space: DCI-P3, Color Temperature [R, G, B]: [100, 97, 82], Gamma: 2.2, Game Visual: User Mode, Screen Saver: Off, Power Setting: Standard Mode, Lighting Effect: Off, Variable Refresh Rate: Off, Shadow Boost: Off.

For the LG UltraGear LCD we adjusted the display settings to the following: Game Mode: Gamer1, Response Time: Off, Adaptive Sync: Off, Brightness: 42, Contrast: 80, Sharpness: 80, Color Temperature [R, G, B]: [43, 40, 39], Gamma: Mode 1, Energy Saving: off, refresh rate: 144 Hz, Dimming: Off

For the Samsung OLED TV the settings were set to the following: brightness: 50 (max), Contrast: 50 (max), Sharpness: 10, Color: 25, Color Tone (G/R): 0, Color tone: Standard, Shadow-Tracing: 0, Color Space Settings: Normal, Gamma: 2.2, White-Balance → 2 points → RGB Gains: [37, 18, −31], RGB Offsets: [0, 0, 0].
